# Synthesis of Functionalized Arylaziridines as Potential Antimicrobial Agents

**DOI:** 10.3390/molecules190811505

**Published:** 2014-08-04

**Authors:** Arianna Giovine, Marilena Muraglia, Marco Antonio Florio, Antonio Rosato, Filomena Corbo, Carlo Franchini, Biagia Musio, Leonardo Degennaro, Renzo Luisi

**Affiliations:** Department of Pharmacy-Drug Science, University of Bari “A. Moro”, Via E.Orabona 4, Bari 70125, Italy; E-Mails: giovinearianna@gmail.com (A.G.); m.muraglia@libero.it (M.M.); marco.florio@libero.it (M.A.F.); antonio.rosato@uniba.it (A.R.); carlo.franchini@uniba.it (C.F.); bm450@cam.ac.uk (B.M.); leonardo.degennaro@uniba.it (L.D.)

**Keywords:** aziridines, boron compounds, Suzuki-Miyaura, palladium coupling, antibiotics

## Abstract

By using the Suzuki-Miyaura protocol, a simple straightforward synthesis of functionalized 2-arylaziridines has been developed. By means of this synthetic strategy from readily available *ortho*-, *meta*- and *para*-bromophenylaziridines and aryl- or heteroarylboronic acids, new aziridines could be obtained. The cross-coupling reactions occurred without ring opening of the three membered ring. Preliminary results on the antimicrobial activity of the heterosubstituted biaryl compounds have been also included.

## 1. Introduction

The importance of aziridines as useful building blocks in organic chemistry has been extensively recognized over the past decades [1,2,3,4,5,6,7,8]. This three-membered ring heterocycle has been successfully exploited as precursors of amino acids [9,10], amino alcohols [11], azomethine ylides [12,13], monomer for polymerization [14], chiral auxiliary [15] and chiral ligand [16,17,18,19,20,21,22,23]. Aziridines are also interesting structural fragments in bioactive compounds and their pharmacological activity is often strictly related to the reactivity of the spring-loaded aziridine functionality [24,25,26], for example by endogenous nucleophiles. Due to their extensive use in organic and medicinal chemistry, several methodologies for the preparation of useful functionalized aziridines have been developed.

**Scheme 1:**
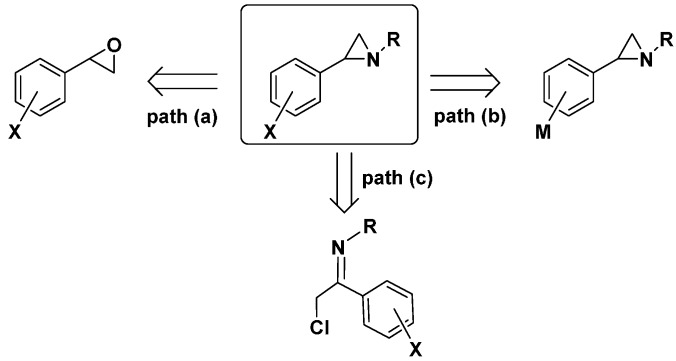
Reaction pathways for the synthesis of *N*-alkylarylaziridines.

In a research work aimed at investigating the reactivity of arylaziridines, we embarked in the preparation of *N*-alkyl-2-arylaziridines functionalized on the aromatic ring. We envisaged two main paths for the functionalization of the aromatic ring depicted in Scheme 1. In particular, *N*-alkyl-2-arylaziridines could be obtained from readily available 2-aryloxiranes (path a) by a ring opening reaction, using primary amine, followed by a cyclization under Mitsunobu conditions [27]. However, the low yields observed with some substituted aryloxiranes, and the poor availability of the corresponding styrenes to be oxidized, limit this synthetic route. Alternatively, the reduction of readily available a-chloro imines also furnishes *N*-alkyl-2-arylaziridines (Scheme 1, path c) [28].Another synthetic strategy (Scheme 1, path b) relies on the use of aziridines metallated on the aromatic ring that can be functionalized with electrophiles or by a cross-coupling strategy [29].

**Scheme 2:**
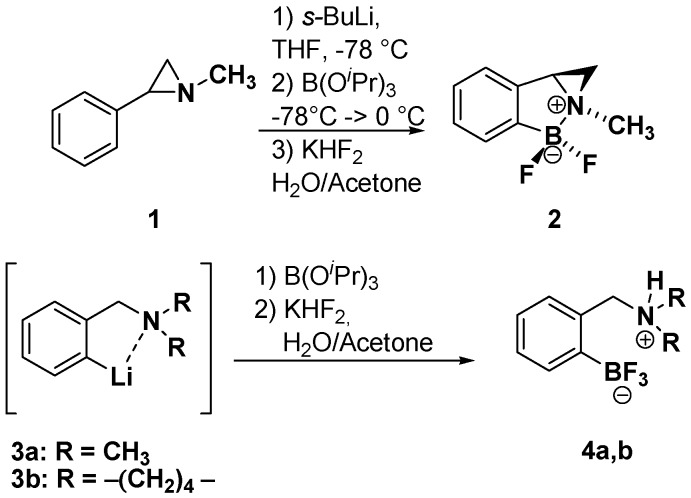
Lithiation-borylation sequence of 2-arylaziridines and benzylamines.

In previous works we have reported that nitrogen-bearing small heterocycles, such as aziridine and azetidines, could promote the direct C-H *ortho*-metallation (lithiation) of the aryl ring [30,31], acting as a directing metallation group (DMG) [32,33]. The synthetic utility of such *ortho*-lithiated intermediates has been widely demonstrated even in a D*o*M-cross coupling strategy [34]. As a matter of fact, a new Suzuki-Miyaura reagent, the cyclic aziridinedifluoroborate **2**, has been prepared from the *N*-methyl-2-phenylaziridine **1** (Scheme 2) [35]. It has been also demonstrated that the cyclic difluoroborate could be obtained only in the lithiation-borylation of 2-arylaziridines, while the use of *ortho*-lithiated benzylamines **3a**,**b** led to the corresponding trifluorobrates **4a**,**b** (Scheme 2) [35].

With the aim to verify this “*ortho*-effect” of the aziridine ring and set-up a procedure for the preparation of functionalized arylaziridines, we investigated the lithiation-borylation sequence in the *meta* and *para* positions of the aromatic ring of *N*-alkyl-2-arylaziridines. The results of this investigation are reported here, jointly to preliminary data on the potential antibacterial and antifungal profile against different bacterial and fungal strains belonging to American Type Culture Collection (ATCC) of *ortho*-, *meta*- and *para*- substituted *N*-alkyl-2-arylaziridines obtained by a Pd-mediated cross-coupling methodology.

## 2. Results and Discussion

### 2.1. Chemistry

**Scheme 3:**
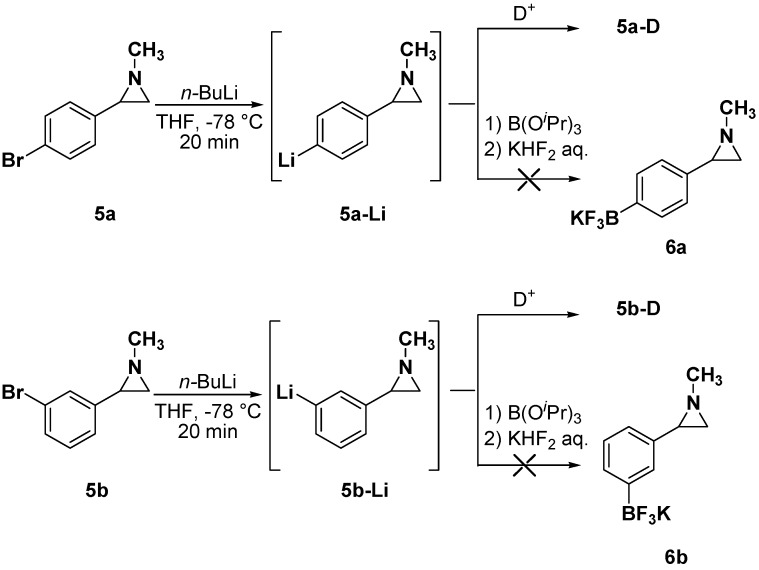
Attempts for lithiation-borylation sequence by bromine-lithium permutation.

In order to obtain the *meta*- and *para*- substituted boron derivatives, the bromine-lithium exchange on *N*-alkyl-2-arylaziridines **5a**,**b** [36,37] was firstly considered (Scheme 3). The bromine-lithium permutation with *n*-BuLi at −78 °C in THF for 20 minutes on aziridines **5a**,**b** generated the corresponding lithiated arylaziridines **5a**,**b**–**Li**, as proved by quenching with a deuterium source affording **5a**–**D** and **5b**–**D** (>98% D).

However, all the attempts to obtain the *meta*- and *para*- boron derivatives by borylation of the lithiated intermediates failed, and only traces of the desired trifluoroborates **6a**,**b** were detected by ^11^B-NMR (Scheme 3). Nevertheless, with brominated arylaziridines **5a**,**b** in hand, we decided to use them as reactants in Suzuki-Miyaura reactions with promptly available aryl and heteroarylboronic acid derivatives. For the sake of comparison, the reactivity of the *ortho*-brominated aziridine **5c** [30,31], which should complement the reactivity of difluoroborate **2**, was also investigated.

**Scheme 4:**
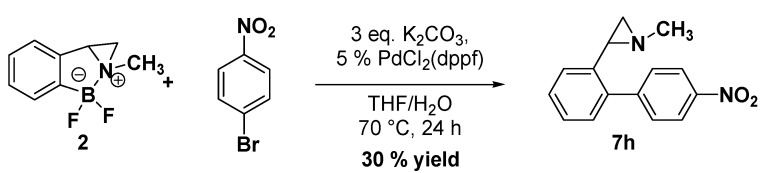
Cross-coupling of aziridinedifluoroborate **2**.

In Table 1 are collected the results for the cross-coupling reactions of bromoarylaziridines **5a**–**c** carried out using different boronic acids (1.5 equiv.), PdCl_2_ (dppf) as the catalyst, K_2_CO_3_ as the base (3 equiv.) in a mixture of THF/H_2_O (90/10) as the reaction solvent at 70 °C for 24 h. By using this procedure *ortho*-, *meta*- and *para*-functionalized arylaziridines **7a**–**i** were obtained. It is worth noting that the reactions proceeded smoothly without aziridine ring opening with good yields in almost all cases. A very low yield was observed only in the coupling reaction of **5c** with *p*-nitrophenylboronic acid to give **7h**. However, the yield of **7h** was improved by using aziridinedifluoroborate **2** and 4-bromonitrobenzene under the reaction conditions reported in the Scheme 4.

**Table 1:** Suzuki-Miyaura reactions of bromoarylaziridines **5a**–**c**. 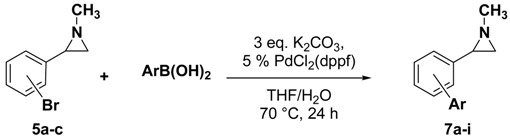

Aziridine 5	ArB(OH)_2_	Product 7	Yield ^a^ (%) ^b^
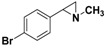 **5a**	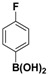	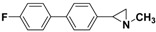 **7a**	80 (41)
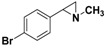 **5a**	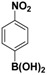	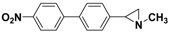 **7b**	80 (72)
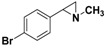 **5a**	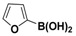	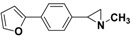 **7c**	80 (48)
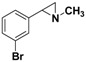 **5b**	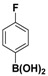	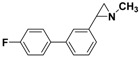 **7d**	85 (70)
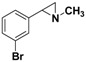 **5b**	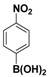	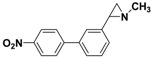 **7e**	73 (34)
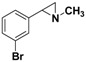 **5b**	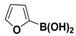	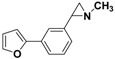 **7f**	85 (77)
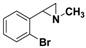 **5c**	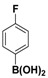	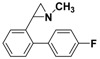 **7g**	(40)
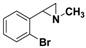 **5c**	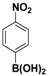	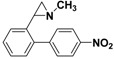 **7h**	(5)
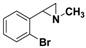 **5c**	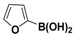	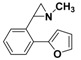 **7i**	65 (48)

*^a^* ^1^H-NMR yield; *^b^* Isolated yield.

It is worth mentioning that, to our knowledge, this is the first example of cross-coupling reactions involving haloarylaziridines [38,39]. By using this strategy, a series of *ortho*-, *meta*- and *para-*functionalized arylaziridines could be prepared.

**Figure 1:**
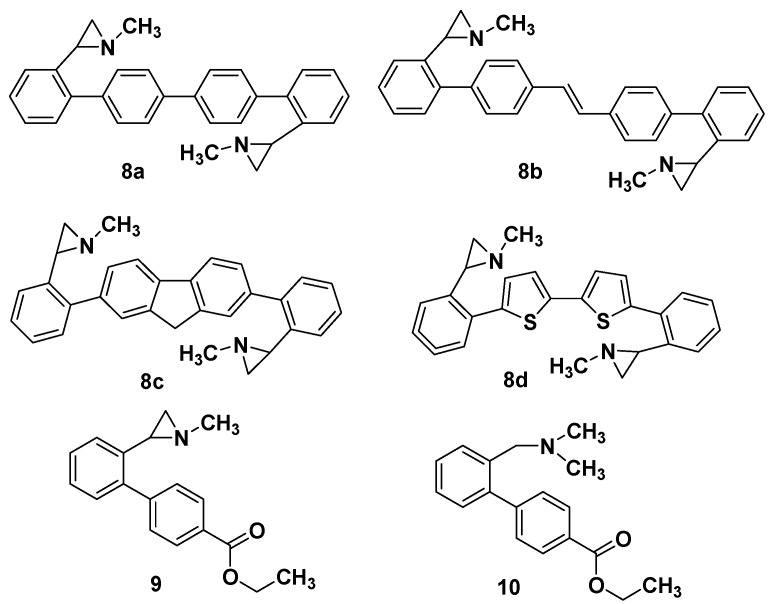
Other functionalized aziridines tested in this study.

With this series of aziridines in hand, and aware that many aziridine alkaloids have antimicrobial activity against selected pathogenic microorganisms [40,41,42,43], we decided to test preliminary all the aziridine derivatives described in this paper for a potential antibacterial and antifungal profile against different bacterial and fungal strains belonging to American Type Culture Collection (ATCC). For the biological evaluation, also the bisaziridines **8a**–**d** and derivatives **9**,**10** were considered (Figure 1) [35].

### 2.2. Biology

The biological data reported in Table 2 revealed that the majority of the evaluated aziridine derivatives exhibited moderate to good activity (MIC: 16–256 µg/mL) as compared to reference antimicrobial drugs.

Among the mentioned derivatives the most promising results were obtained with compounds **8a**, **8c**, **9**, **7e**, **7d**, **7a** and **2**, bearing the aziridinic moiety. In this series, compounds **2** and **8a** were the most active derivatives. In particular, **8a** showed both a selective antibacterial activity against *Enterococcus faecalis* 29212 (MIC: 16 µg/mL) and an interesting antifungal action against *Candida krusei* 6258 (MIC: 16 µg/mL). An appreciable antibacterial profile against bacteria such as *Staphylococcus aureus* 29213 and *Enterococcus faecalis* 29212 with MIC values of 32 µg/mL and 16 µg/mL, respectively, was registered for compound **2**. In addition, it is worth noting that compounds **8a**, **8c**, **7d** and **7a** revealed a significant broad spectrum of action.

Due to both the limited number of evaluated compounds, and the low diversity of the involved chemical features of the series herein reported, an attempt to analyze the structure-activity relationship (SAR) does not seem reasonable. Nevertheless, several comparisons on the results could be made.

**Table 2:** Antimicrobial activity results ^a, b^ of functionalized aziridines derivatives **2**, **4a**,**b**, **7a**–**i**, **8a**–**d**, **9**, **10** (MIC in µg/mL).

Microorganism							
Compd	Bacterial strains			Fungal strains			
	*S.* *aureus* ^c^ 29213	*E. faecalis* ^c^ 29212	*E.* *coli* ^c^ 25922	*C. albicans* ^c^ 10231	*C. parapsilosis* ^c^ 22019	*C. tropicalis* ^c^ 750	*C. krusei* ^c^ 6258	
8a	128	16	>128	64	32	32	16	
8b	>128	>128	>128	R	R	R	R	
8d	256	256	>256	>128	>128	>128	>128	
8c	64	64	>128	>128	32	64	32	
7c	>128	>128	>128	>128	>128	128	128	
7f	128	128	>128	>128	64	128	128	
7i	128	128	>128	>128	128	>128	>128	
9	128	32	>128	>128	128	128	>128	
10	>128	>128	>128	>128	>128	>128	>128	
7b	128	>128	>128	>128	>128	128	128	
7e	64	128	>128	>128	128	128	128	
7d	64	64	>128	128	64	128	64	
7h	128	128	>128	>128	128	128	128	
7g	128	128	>128	>128	128	128	128	
7a	64	64	>128	128	128	128	64	
2	32	16	>128	>128	>128	>128	>128	
4a	>128	>128	>128	>128	>128	>128	>128	
4b	>128	>128	>128	>128	>128	>128	>128	
NRF ^c^	2	4	0.03					
CAF ^c^	8	4	8					
OXA ^c^	0.250	16	R					
FLU ^c^				0.5		1		
AMB ^c^				0.5	1	1	1	

*^a^* National Committee for Clinical Laboratory Standards. Methods for Dilution Antimicrobial Susceptibility Tests for Bacteria that Grow Aerobically, Sixth Edition. Approved Standard NCCLS Document M7-A6, Vol. 23, No. 2 NCCLS, Wayne, PA, January, 2003; *^b^* National Committee for Clinical Laboratory Standards. Reference Methods for Broth Dilution Antifungal Susceptibility Testing of Yeast. Approved Standard, 2nd ed. M27-A2, Vol. 22, No. 15 NCCLS, Wayne, PA, January, 2002; *^c^ S. aureus*: *Staphylococcus aureus*; *E. faecalis*: *Enterococcus faecalis*; *E.coli*: *Escherichia coli*; *C. albicans*: *Candida albicans*; *C. parapsilosis*: *Candida parapsilosis*; *C. tropicalis*: *Candida tropicalis*; *C. krusei*: *Candida krusei*; R: resistant; NRF: Norfloxacin; CAF: Chloroamphenicol; OXA: Oxacillin; FLU: Fluconazole; AMB: Amphotericin B.

First, the SAR analysis suggested that the *N*-methylaziridinyl group plays a noticeable role for producing an antimicrobial profile. In fact, replacing the aziridine ring with a dimethylamine or proline moiety, a marked reduction of the antimicrobial activity was observed (Table 2, see data **9** *vs*. **10** and **2** *vs*. **4a** or **4b**).

Interestingly, the MIC values of compound **2**, ranging between 16 and 32 µg/mL, could likely be attributed to the combined action of the aziridine ring and the BF2 fragment respectively at C2 and C1 positions of the aromatic ring.

Among the biaryl derivatives **7** (Table 1) the replacement of the nitro group with a fluorine atom produced an enlargement of the antimicrobial spectrum (**7e** *vs.* **7d**). In the same series, the change in the position of the aziridine ring from *ortho* to *meta* to *para* on the aromatic ring, generally was found to be important for enhancing the antimicrobial activity.

All the above considerations prompted us to take into accountalso bisaziridinyl derivatives such as compounds **8a**–**d**. With reference to this last series, the biological data suggested that the insertion of an additional aziridine ring as in **8a**, gave still a wide antifungal spectrum of action and a selective antibacterial profile against *Enterococcus faecalis* 29212. However, it was further observed that the substitution of the 4,4'-diphenyl core of **8a** with the 4,4’-stilbene unit or the 5,5'-bisthienyl moiety resulted in a significant decreasing of the antimicrobial activity (Table 2, **8a** *vs. ***8b** and **8d**). On the other hand the introduction of a rigidity element with the insertion of a 2,7-fluorenyl portion as in **8c**, led to a wider spectrum of action.

However, these preliminary results showed that among all the tested aziridines, **2** and **8a** were identified as the most interesting compounds with a remarkable antimicrobial profile. According to the studies herein described, we could point out that the aziridine ring likely could play an important role in the antimicrobial activity.

## 3. Experimental Section

### 3.1. Chemistry

Commercial reagents were purchased from Sigma–Aldrich (St. Louis, MO, USA), Alfa Aesar (Ward Hill, MA, USA) and were used without further purification unless otherwise noted. Tetrahydrofuran (THF) was freshly distilled under nitrogen over sodium/benzophenone ketyl. Water was deionized prior to use. Magnetic Resonance spectra were recorded using Varian 400 and 500 MHz (Cernusco s/N, MI, Italy) and Bruker 400, 500 and 600 MHz spectrometers (Milano, Italy,). For the ^1^H and ^13^C-NMR spectra (^1^H-NMR 400, 500, 600 MHz and ^13^C-NMR 100, 125, 150 MHz) CDCl_3_was used as solvent, using tetramethylsilane as internal standard and chemical shifts are reported as part per million (ppm). MS-ESI analyses were performed on LC/MSD trap system VL (Cernusco s/N, MI, Italy). GC-MS spectrometry analyses were carried out on a gas chromatograph (dimethylsilicon capillary column, 30 m, 0.25 mm i.d.) equipped with a mass selective detector operating at 70 eV (EI) (Cernusco s/N, MI, Italy). Analytical thin layer chromatography (TLC) was carried out on precoated 0.25 mm thick plates of Kieselgel 60 F254; visualization was accomplished by UV light (254 or 356 nm) or by spraying a solution of 5% (w/v) ammonium molybdate and 0.2% (w/v) cerium(III) sulphate in 100 mL 17.6% (w/v) aq. sulphuric acid and heating to 200 °C for some time until blue spots appear. Infrared spectra of the compounds were recorded neat, as film or as KBr disc as indicated, by a Perkin-Elmer 283 spectrometer (Waltham, MA, USA). For flash chromathography silica Gel 60, 0.04–0.063 mm particle size was used. CHN analyses were performed on a EuroEA 3000 analyzer (Milano, Italy).

### 3.2. General Procedure for the Synthesis of Aziridine **7**

To a degassed solution of *N*-methyl-2-(bromophenyl)aziridine (106 mg, 0.5 mmol) in THF/H_2_O (90:10) (5 mL) were added arylboronic acid (0.75 mmol), K_2_CO_3_ (207 mg, 1.5 mmol) and [1,1′-Bis(diphenylphosphino)ferrocene]dichloropalladium(II), (PdCl_2_(dppf)∙CH_2_Cl_2_)_,_ (20 mg, 0.025 mmol). The reaction mixture was stirred at 70 °C, until aziridine had been completely consumed as determined by TLC or GC-MS. The reaction mixture was allowed to cool to room temperature and diluted with water (10 mL) followed by extraction with Et_2_O (15 mL × 3). The solvent was removed *in vacuo*, and the crude product was purified by silica gel column chromatography to yield the pure product.

*2-(4'-Fluorobiphenyl-4-yl)-1-methylaziridine* **7a**. The general procedure was followed using *N*-methyl-2-(4-bromophenyl)aziridine **5a** (106 mg, 0.5 mmol) and 4-fluorophenylboronic acid (105 mg, 0.75 mmol). Flash chromatography (Hexane/Et_2_O 10:90) yielded **7a** (41%, 47 mg, 0.205 mmol) as a white solid (m.p. 84 °C,). ^1^H-NMR (600 MHz, CDCl_3_) *δ* 1.69 (d, *J* = 6.4 Hz, 1H, CH_2_), 1.96 (d, *J* = 3.2 Hz, 1H, CH_2_), 2.33 (dd, *J* = 6.5, 3.2 Hz, 1H, CH), 2.53 (s, 3H, CH_3_), 7.11–7.14 (m, 2H, ArH), 7.31 (m, 2H, ArH), 7.49 (m, 2H, ArH), 7.52–7.55 (m, 2H, ArH). ^13^C-NMR (100 MHz, CDCl_3_) *δ* 39.4, 42.0, 47.8, 115.5 (d, ^2^*J* (C-F) = 21.5 Hz), 126.4, 126.8, 128.45 (d, ^3^*J* (C-F) = 8.0 Hz), 137.0 (d, ^4^*J* (C-F) = 3.4 Hz), 138.8, 139.4, 162.3 (d, ^1^*J* (C-F) = 246.7 Hz). FT-IR (KBr, cm^−1^) *υ* 3041, 2974, 2951, 2918, 2852, 2784, 1601, 1497, 1450, 1383,1237, 1213, 1161, 1110, 1070, 1013, 828. GC-MS (70 eV) *m/z (%)* 227(28, M^+^), 226 (100, M-H^+^), 185 (15), 183 (21). Anal. Calcd (%) for C_15_H_14_FN: C, 79.27; H, 6.21; N, 6.16. Found: C, 79.18; H, 6.29; N, 6.08.

*2-(4'-Nitrobiphenyl-4-yl)-1-methylaziridine* **7b**. The general procedure was followed using *N*-methyl-2-(4-bromophenyl)aziridine **5a** (106 mg, 0.5 mmol) and 4-nitrophenylboronic acid (125 mg, 0.75 mmol). The crude mixture was suspended in hexane and then filtered. The filtrate was concentrated *in vacuo* to yield **7b** as an orange waxy solid (72%, 91 mg, 0.36 mmol). ^1^H-NMR (400 MHz, CDCl_3_) *δ* 1.68 (d, *J* = 6.5 Hz, 1H, CH_2_), 1.91 (d, *J* = 3.3 Hz, 1H, CH_2_), 2.31 (dd, *J* = 6.5, 3.3 Hz, 1H, CH), 2.49 (s, 3H, CH_3_), 7.32 (m, 2H, ArH), 7.53 (m, 2H, ArH), 7.68 (m, 2H, ArH), 8.25 (m, 2 H, ArH). ^13^C-NMR (100 MHz, CDCl_3_) *δ* 39.6, 41.9, 47.8, 124.1, 126.8, 127.2, 127.5, 137.2, 141.5, 146.9, 147.3. FT-IR (film, cm^−1^) *υ* 2963, 1596, 1513, 1447, 1400, 1338, 1260, 1094, 1019, 864, 800, 696. GC-MS (70 eV) *m/z (%)* 254 (25, M^+^), 253 (100, M-H^+^), 207 (26), 165 (24). Anal. Calcd (%) for C_15_H_14_N_2_O_2_: C, 70.85; H, 5.55; N, 11.02; O, 12.58. Found: C, 70.80; H, 5.59; N, 11.05; O, 12.57.

*2*-[4-(Furan-2-yl)-phenyl]-*1-methylaziridine* **7c**. The general procedure was followed using *N*-methyl-2-(4-bromophenyl)aziridine **5a** (106 mg, 0.5 mmol) and 2-furanylboronic acid (84 mg, 0.75 mmol). Flash chromatography (Hexane/EtOAc 50:50) yielded **7c** as a yellow oil (48%, 48 mg, 0.24 mmol).^1^H-NMR (400 MHz, CDCl_3_) *δ* 1.61 (d, *J* = 6.4 Hz, 1H, CH_2_), 1.88 (d, *J* = 3.3 Hz, 1H, CH_2_), 2.24 (dd, *J* = 6.4, 3.3 Hz, 1H, CH), 2.46 (s, 3H, CH_3_), 6.41–6.43 (m, 1H, ArH), 6.58–6.57 (m, 1H, ArH), 7.20 (m, 2H, ArH), 7.41 (m, 1H, ArH), 7.57 (m, 2 H, ArH). ^13^C-NMR (100 MHz, CDCl_3_) *δ* 39.4, 42.2, 47.9, 104.6, 111.6, 123.7, 126.3, 129.6, 139.4, 141.8, 153.9. FT-IR (film, cm^−1^) *υ* 3114, 3042, 2973, 2945, 2849, 2780, 1518, 1482, 1450, 1412, 1385, 1278, 1242, 1217, 1186, 1156, 1112, 1078, 1008, 903, 884, 846, 782, 766, 733, 667. GC-MS (70 eV) *m/z (%)* 199 (40, M^+^), 198 (100, M-H^+^), 157 (20), 128 (19). Anal. Calcd (%) for C_13_H_13_NO: C, 78.36; H, 6.58; N, 7.03; O, 8.03. Found: C, 78.29; H, 6.56; N, 6.99; O, 8.07.

*2-(4'-Fluorobiphenyl-3-yl)-1-methylaziridine* **7d**. The general procedure was followed using *N*-methyl-2-(3-bromophenyl)aziridine **5b** (106 mg, 0.5 mmol) and 4-fluorophenylboronic acid (105 mg, 0.75 mmol). Flash chromatography (Hexane/Et_2_O 10:90) yielded **7d** as a yellow oil (70%, 80 mg, 0.35 mmol). ^1^H-NMR (400 MHz, CDCl_3_) *δ* 1.65 (d, *J* = 6.5 Hz, 1 H, CH_2_), 1.93 (d, *J* = 3.3 Hz, 1 H, CH_2_), 2.31 (dd, *J* = 6.5, 3.3 Hz, 1 H, CH), 2.49 (s, 3 H, CH_3_), 7.06–7.11 (m, 2 H, ArH), 7.18–7.20 (m, 1 H, ArH), 7.32–7.39 (m, 3 H, ArH), 7.50–7.55 (m, 2 H, ArH). ^13^C-NMR (100 MHz, CDCl_3_) *δ* 39.5, 42.3, 48.0, 115.5 (d, ^2^*J* (C-F) = 21.4 Hz), 124.6, 125.0, 125.6, 128.7 (d, ^3^*J* (C-F) = 8.0 Hz), 128.8 (s overlapping d), 137.2 (d, ^4^*J* (C-F) = 3.2 Hz), 140.3, 140.9, 162.5 (d, ^1^*J* (C-F) = 246.7 Hz). FT-IR (film, cm^−1^) *υ* 3044, 2946, 2849, 1608, 1514, 1483, 1450, 1400, 1367, 1224, 1180, 1158, 1096, 1070, 1013, 837, 807, 773, 702. GC-MS (70 eV) *m/z (%)* 227 (26, M^+^), 226 (100, M-H^+^), 183 (21). Anal. Calcd (%) for C_15_H_14_FN: C, 79.27; H, 6.21; N, 6.16. Found: C, 79.20; H, 6.25; N, 6.12.

*1-Methyl-2-(4'-nitrobiphenyl-3-yl)aziridine* **7e**. The general procedure was followed using *N*-methyl-2-(3-bromophenyl)aziridine **5b** (106 mg, 0.5 mmol) and 4-nitrophenylboronic acid (125 mg, 0.75 mmol). Flash chromatography (Hexane/EtOAc 40:60) yielded **7e** as a yellow oil (34%, 43 mg, 0.17mmol). ^1^H-NMR (400 MHz, CDCl_3_) *δ* 1.67 (d, *J*= 6.5 Hz, 1H, CH_2_), 1.92 (d, *J* = 3.3 Hz, 1H, CH_2_), 2.34 (dd, *J* = 6.5, 3.3 Hz, 1H, CH), 2.49 (s, 3H, CH3), 7.28 (m, 1H, ArH), 7.36–7.45 (m, 3H, ArH), 7.70 (m, 2H, ArH), 8.24 (m, 2H, ArH). 13C-NMR (100 MHz, CDCl3) δ 39.6, 42.2, 47.9, 124.0, 124.8, 125.9, 126.8, 127.8, 129.1, 138.8, 141.4, 147.0, 147.5. FT-IR (film, cm^−1^) *υ* 3046, 2946, 2850, 2783, 1596, 1515, 1480, 1449, 1400, 1345, 1262, 1243, 1187, 1107, 1070, 1013, 961, 854, 802, 777, 751, 696. GC-MS (70 eV) *m/z (%)* 254 (26, M^+^), 253 (100, M-H^+^), 207 (29), 165 (24). Anal. Calcd (%) for C_15_H_14_N_2_O_2_: C, 70.85; H, 5.55; N, 11.02; O, 12.58. Found: C, 70.87; H, 5.51; N, 11.08; O, 12.61.

*2*-[3-(Furan-2-yl)phenyl]-*1-methylaziridine* **7f**. The general procedure was followed using *N*-methyl-2-(3-bromophenyl)aziridine **5b** (106 mg, 0.5 mmol) and 2-furanylboronic acid (84 mg, 0.75 mmol). Flash chromatography (Hexane/EtOAc 50:50) yielded **7f** as a yellow oil (77%, 77 mg, 0.385mmol). ^1^H-NMR (400 MHz, CDCl_3_) *δ* 1.62 (d, *J* = 6.5 Hz, 1 H, CH_2_), 1.91 (d, *J* = 3.2 Hz, 1 H, CH_2_), 2.27 (dd, *J* = 6.5, 3.2 Hz, 1 H, CH), 2.47 (s, 3 H, CH_3_), 6.42–6.43 (m, 1 H, ArH), 6.62–6.63 (m, 1 H, ArH), 7.09 (m, 1 H, ArH), 7.26–7.30 (m, 1 H, ArH), 7.42 (m, 1 H, ArH), 7.50–7.52 (m, 2 H, ArH). ^13^C-NMR (100 MHz, CDCl_3_) *δ* 39.3, 42.3, 47.9, 105.1, 111.6, 121.3, 122.3, 125.0, 128.6, 130.9, 140.7, 141.9, 153.9. FT-IR (film, cm^−1^) *υ* 2954, 2849, 2782, 1613, 1450, 1386, 1219, 1187, 1155,1068, 1012, 912, 771, 732. GC-MS (70 eV) *m/z (%)* 199 (35, M^+^), 198 (100, M-H^+^), 128 (17). Anal. Calcd (%) for C_13_H_13_NO: C, 78.36; H, 6.58; N, 7.03; O, 8.03. Found: C, 78.31; H, 6.52; N, 7.08; O, 8.09.

*2-(4'-Fluorobiphenyl-2-yl)-1-methylaziridine* **7g**. The general procedure was followed using *N*-methyl-2-(2-bromophenyl)aziridine **5c** (106 mg, 0.5 mmol) and 4-fluorophenylboronic acid (105 mg, 0.75 mmol). Flash chromatography (Hexane/Et_2_O 10:90) yielded **7g** as a yellow oil (40%, 45 mg, 0.2 mmol). ^1^H-NMR (400 MHz, CDCl_3_) *δ* 1.51 (d, *J* = 6.5 Hz, 1H, CH_2_), 1.90 (d, *J* = 3.4 Hz, 1H, CH_2_), 2.21 (dd, *J* = 6.5, 3.4 Hz, 1H, CH), 2.32 (s, 3H, CH_3_), 7.08–7.13 (m, 2H, ArH), 7.18–7.19 (m, 1H, ArH), 7.22–7.30 (m, 3H, ArH), 7.32–7.38 (m, 2H, ArH). ^13^C-NMR (100 MHz, CDCl_3_) *δ* 39.4, 40.6, 47.6, 115.0 (d, ^2^*J* (C-F) = 21.3 Hz), 125.5, 126.6, 127.7, 129.5, 131.1 (d, ^3^*J* (C-F) = 8.0 Hz), 137.0 (d, ^4^*J* (C-F) = 3.3 Hz), 137.4, 140.8, 162.1 (d, ^1^*J* (C-F) = 246.0 Hz). FT-IR (film, cm^−1^) *υ* 3041, 2945, 2849, 2778, 1606, 1513, 1482, 1449, 1413, 1381, 1222, 1158, 1093, 1009, 838, 761, 741. GC-MS (70 eV) *m/z (%)* 227 (24, M^+^), 226 (100, M-H^+^), 184 (42), 183 (90). Anal. Calcd (%) for C_15_H_14_FN: C, 79.27; H, 6.21; N, 6.16. Found: C, 79.31; H, 6.19; N, 6.11.

*2*-[2-(Furan-2-yl)phenyl]-*1-methylaziridine* **7i**. The general procedure was followed using *N*-methyl-2-(2-bromophenyl)aziridine **5c** (106 mg, 0.5 mmol) and 2-furanylboronic acid (84 mg, 0.75 mmol). The crude mixture was suspended in hexane and then filtered. The filtrate was concentrated in vacuo to yield **7i** as a brown oil (48%, 48 mg, 0.24 mmol). ^1^H-NMR (400 MHz, CDCl_3_) *δ* 1.65 (d, *J* = 6.5 Hz, 1H, CH_2_), 1.86 (d, *J* = 3.4 Hz, 1H, CH_2_), 2.48 (s, 3H, CH_3_), 2.63 (dd, *J* = 6.5, 3.4 Hz, 1H, CH), 6.49–6.50 (m, 1H, ArH), 6.63 (m, 1H, ArH), 7.24–726 (m, 2H, ArH), 7.40–7.42 (m, 1H, ArH), 7.51 (m, 1H, ArH), 7.59–7.61 (m, 1H, ArH). ^13^C-NMR (100 MHz, CDCl_3_) *δ* 39.1, 41.3, 47.8, 108.8, 111.3, 126.6, 126.8, 127.1, 127.9, 130.0, 136.6, 142.0, 153.1. FT-IR (film, cm^−1^) *υ* 3056, 2965, 2850, 1483, 1450, 1262, 1156, 1081, 1051, 1029, 1010, 803, 761, 735. GC-MS (70 eV) *m/z (%)* 199 (46, M^+^), 198 (78, M-H^+^), 182 (23), 170 (100), 144 (33), 128 (32). Anal. Calcd (%) for C_13_H_13_NO: C, 78.36; H, 6.58; N, 7.03; O, 8.03. Found: C, 78.40; H, 6.49; N, 7.06; O, 7.98.

### 3.3. Synthesis of 1-Methyl-2-(4'-nitrobiphenyl-2-yl)aziridine **7h**

To a degassed solution of aziridinedifluoroborate 2 (127 mg, 0.7 mmol) in THF/H_2_O (90:10) (10 mL) were added K_2_CO_3_ (290 mg, 2.1 mmol), 1-bromo-4-nitrobenzene (141 mg, 0.7 mmol) and PdCl_2_(dppf)∙CH_2_Cl_2_ (28 mg, 0.035 mmol). The reaction mixture was stirred at 70 °C for 24 h, was allowed to cool to room temperature, diluted with water (10 mL) and extracted with Et_2_O (15 mL ×3). The solvent was removed *in vacuo* and the crude mixture was purified by flash chromatography on silica gel (Hexane/EtOAc 50:50) to yield aziridine **7h** (30%, 53 mg, 0.21 mmol) as a pale brown solid (m.p. 74 °C). ^1^H-NMR (400 MHz, CDCl_3_) *δ* 1.53 (d, *J* = 6.5 Hz, 1 H, CH_2_), 1.90 (d, *J* = 3.3 Hz, 1 H, CH_2_), 2.14 (dd, *J* = 6.5, 3.3 Hz, 1 H, CH), 2.31 (s, 3 H, CH_3_), 7.19–7.35 (m, 4 H, ArH), 7.57 (m, 2 H, ArH), 8.26 (m, 2 H, ArH).^13^C-NMR (100 MHz, CDCl_3_) *δ* 39.5, 40.4, 47.6, 123.4, 126.0, 126.9, 128.3, 128.8, 129.2, 130.5, 137.3, 130.4, 148.0. FT-IR (film, cm^−1^) *υ* 3059, 2946, 2850, 2779, 1596, 1515, 1478, 1449, 1381, 1347, 1313, 1241, 1201, 1185, 1103, 1071, 1008, 855, 802, 770, 750, 700. GC-MS (70 eV) *m/z (%)* 254 (26, M^+^), 253 (100, M-H^+^), 207 (22), 165 (53), 164 (26). Anal. Calcd for C_15_H_14_N_2_O_2_: C, 70.85; H, 5.55; N, 11.02; O, 12.58. Found: C, 70.83; H, 5.54, N, 11.09; O, 12.53.

### 3.4. Biology

#### 3.4.1. Test Organisms

Three bacteria strains available as freeze-disces, belonging to ATCC collection were used: Gram-positive such as *Staphylococcus aureus* ATCC 29213 and *Enterococcus faecalis* ATCC 29212, and Gram-negative *Escherichia col*i ATCC 25922.

Four yeast strains (ATCC) were used: *Candida albicans* 10231, *Candida parapsilosis* 22019, *Candida tropicalis* 750 and *Candida Krusei* 6258. To preserve the purity of cultures and to ensure the reproducibility, it was set up a series of criovials of all microbial strains in glycerolic medium and stored at −80 °C.

The *in vitro* Minimum Inhibitory Concentrations (MICs, μg/mL) of the prepared compounds were carried out by the broth microdilution method, using a 96 well plates (Microtiter^®^), according to the National Committee for Clinical Laboratory Standards (CLSI) formerly NCCLS [44].

Stock solutions of the tested compounds **2**; **4a**,**b**; **7a**–**i**; **8a**–**d**; **9**; **10** were obtained in DMSO. Stock solutions of lower concentrations were prepared for the substances which did not dissolve well. Then two-fold serial dilutions in the suitable starting from 256 μg/mL were plated. In each well 200 μL of these solutions was added. To be sure that no adverse effect on bacterial growth could be caused by the solvent, a control test was carried out by using DMSO at its maximum concentration along with the medium.

#### 3.4.2. Antibacterial Activity

*Pre*-cultures of each bacterial strain were prepared in Cation Adjusted Muller-Hinton broth (CAMHB, Merck–Darmstadt, Germany) and incubated at 37 °C until the growth was complete. The absorbance of these cellular suspensions calibrated at a wavelength of 625 nm using spectrophotometric method (Thermo Spectronic, Genesis 20, Waltham, MA, USA), should range between 0.08 and 0.10 for the 0.5 McFarland standard, corresponding approximately to 108 CFU/mL.

Further the standardized suspension was diluted 1:100 with CAMHB to have 1–2 × 10^6^100 CFU/mL. Every well were seeded with 100 µL of inoculums. The plates were incubated at 37 °C ± 1 for 24 h, and the MIC values recorded as the last well containing no bacterial growth. A number of wells containing only inoculated broth as control growth were prepared. Norfloxacin, Chloramphenicol and Oxacillin were used as standard drugs.

#### 3.4.3. Antifungal Activity

*Pre*-cultures of each yeast strain were prepared in Sabouraud broth 2% glucose (SAB), and incubated at 35 °C ± 1 until the growth was complete. The turbidity of yeast stock was calibrated to 0.5 McFarland standard by spectrophotometric method (530 nm, absorbance range 0.12–0.15) and further the standardized suspension was diluted firstly 1:50 with SAB and then 1:20 in the same medium to have 1–5 × 10^6^CFU/mL [45].

Every well was seeded with 100 µL of inoculums. The plates were incubated at 35 °C ± 1 for 24–48 h, and the MIC values recorded as the last well containing no fungal growth. Amphotericin B and Fluconazole were used as standard drugs.

## 4. Conclusions

In conclusion, we reported the synthesis and a preliminary antimicrobial evaluation of novel functionalized arylaziridines. The synthetic strategy relies on the cross-coupling reactions between bromoarylaziridines and boronic acids. A series of *ortho*-, *meta*- and *para*-functionalized arylaziridines have been synthesized. 

In addition, all the collected data could furnish very valuable information in order to design and synthesize new, versatile and attractive motifs potentially useful in the antimicrobial research investigation.

## Supplementary Materials

Supplementary materials can be accessed at: http://www.mdpi.com/1420-3049/19/8/11505/s1.

## Supplementary Files

Supplementary File 1
