# Supplementary material for: Synthesis of Functionalized Arylaziridines as Potential Antimicrobial Agents
Source: Molecules. 2014 Aug 4;19(8):11505–19. doi: 10.3390/molecules190811505 (PMC6271868; doi:10.3390/molecules190811505)
Supplement: Supplementary File 1 [file molecules-19-11505-s001.pdf]

Supplementary Data

Synthesis of Functionalized Arylaziridines as Potential Antimicrobial Agents

Arianna Giovine, Marilena Muraglia, Marco Antonio Florio, Antonio Rosato, Filomena Corbo *, Carlo Franchini, Biagia Musio, Leonardo Degennaro and Renzo Luisi *

Department of Pharmacy-Drug Science, University of Bari "A. Moro", Via E.Orabona 4, Bari 70125, Italy

Contents

^{1}H- and ^{13}C-NMR Spectra of Aziridines 7a–i

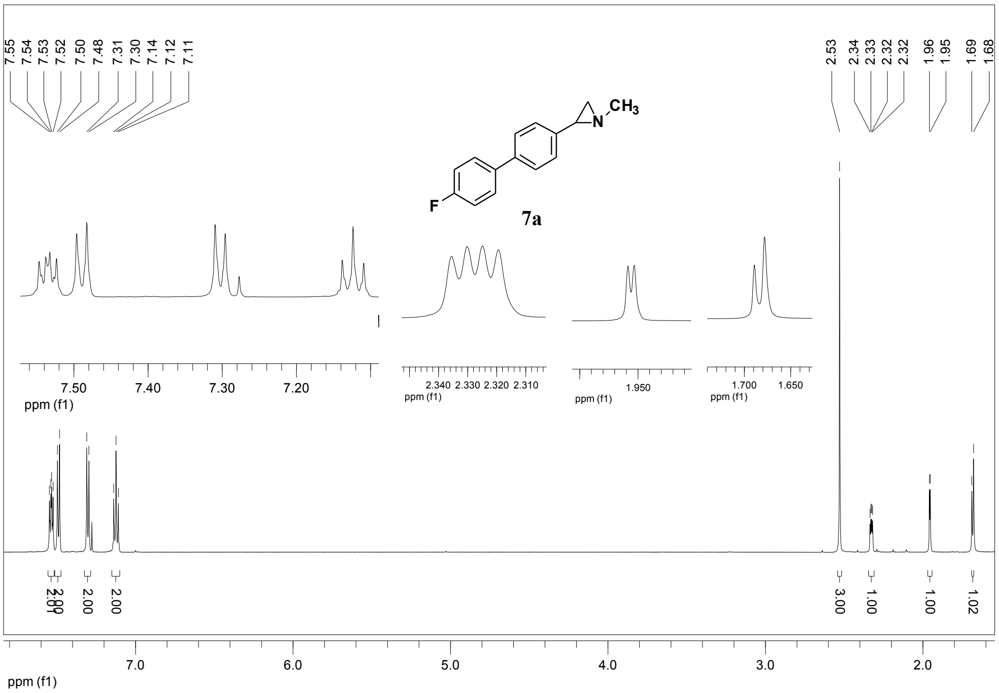

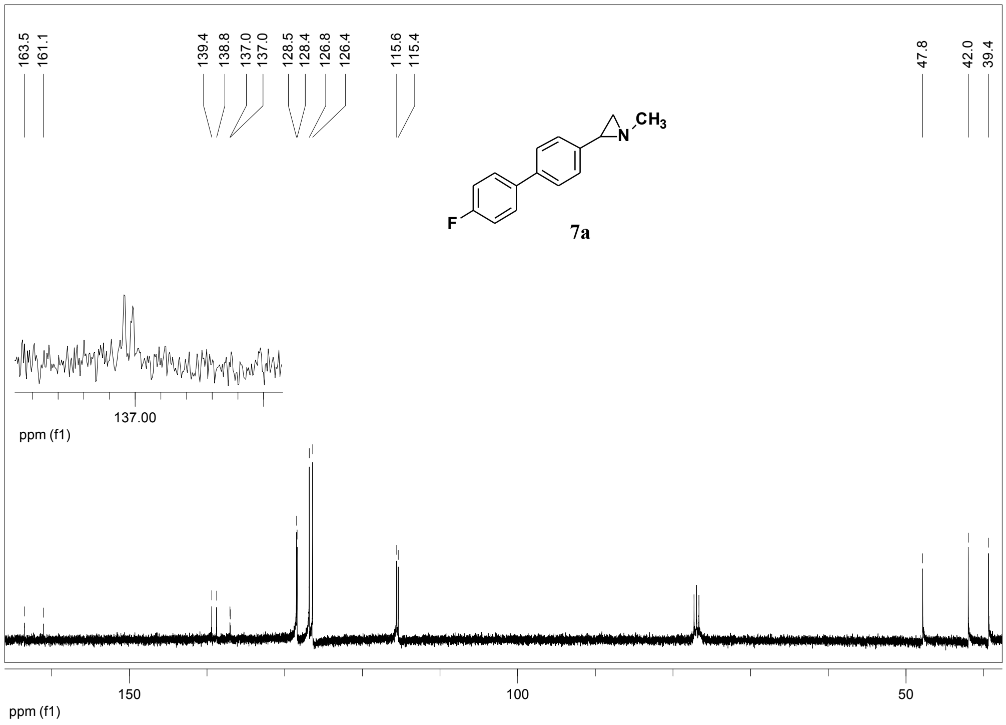

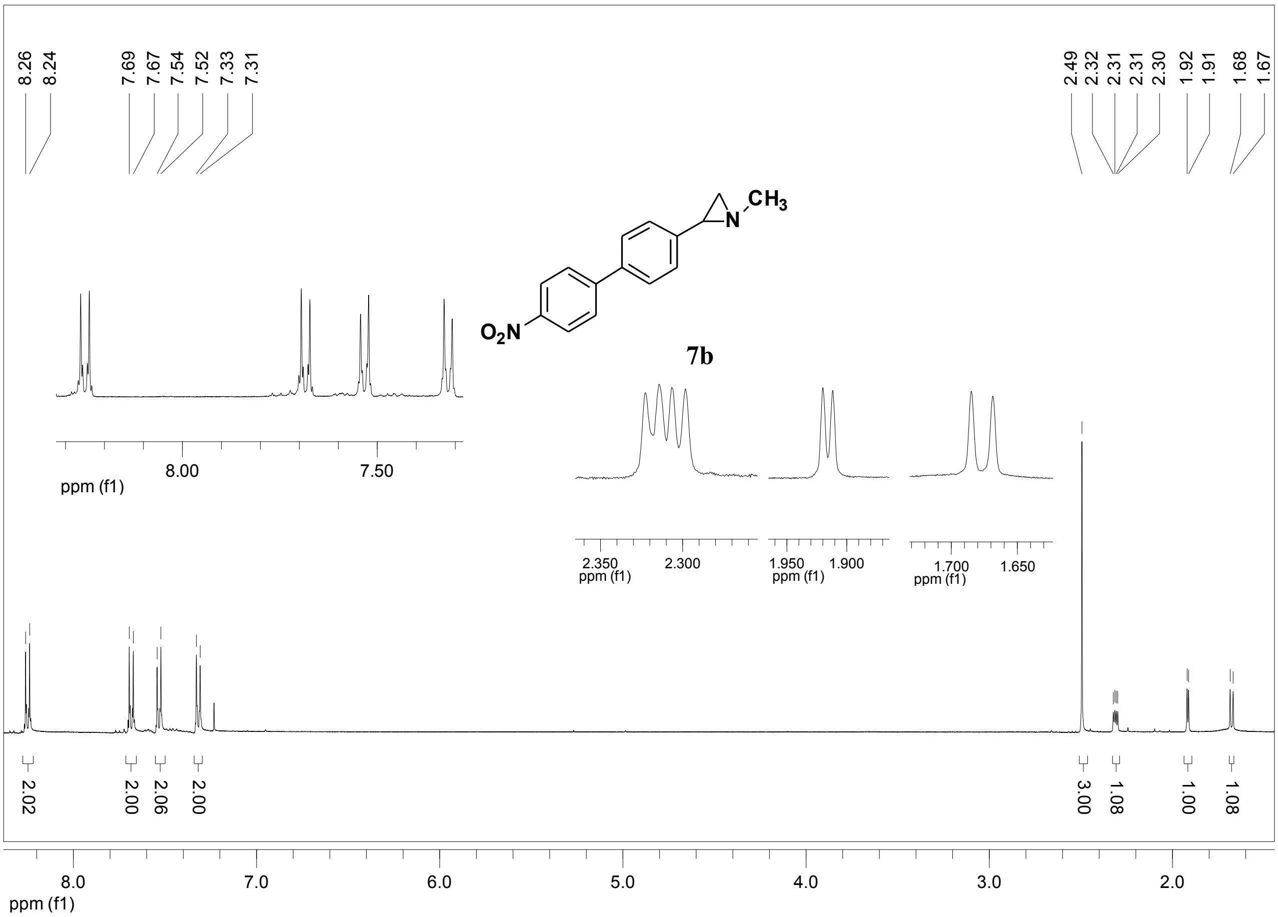
8.26
8.24
7.69
7.67
7.54
7.52
7.33
7.31
2.49
2.32
2.31
2.31
2.30
1.92
1.91
1.68
1.67
CH3
N
O2N
7b
ppm (f1)
8.00
7.50
2.350
2.300
1.950
1.900
1.700
1.650
2.02
2.00
2.06
2.00
3.00
1.08
1.00
1.08
8.0
7.0
6.0
5.0
4.0
3.0
2.0

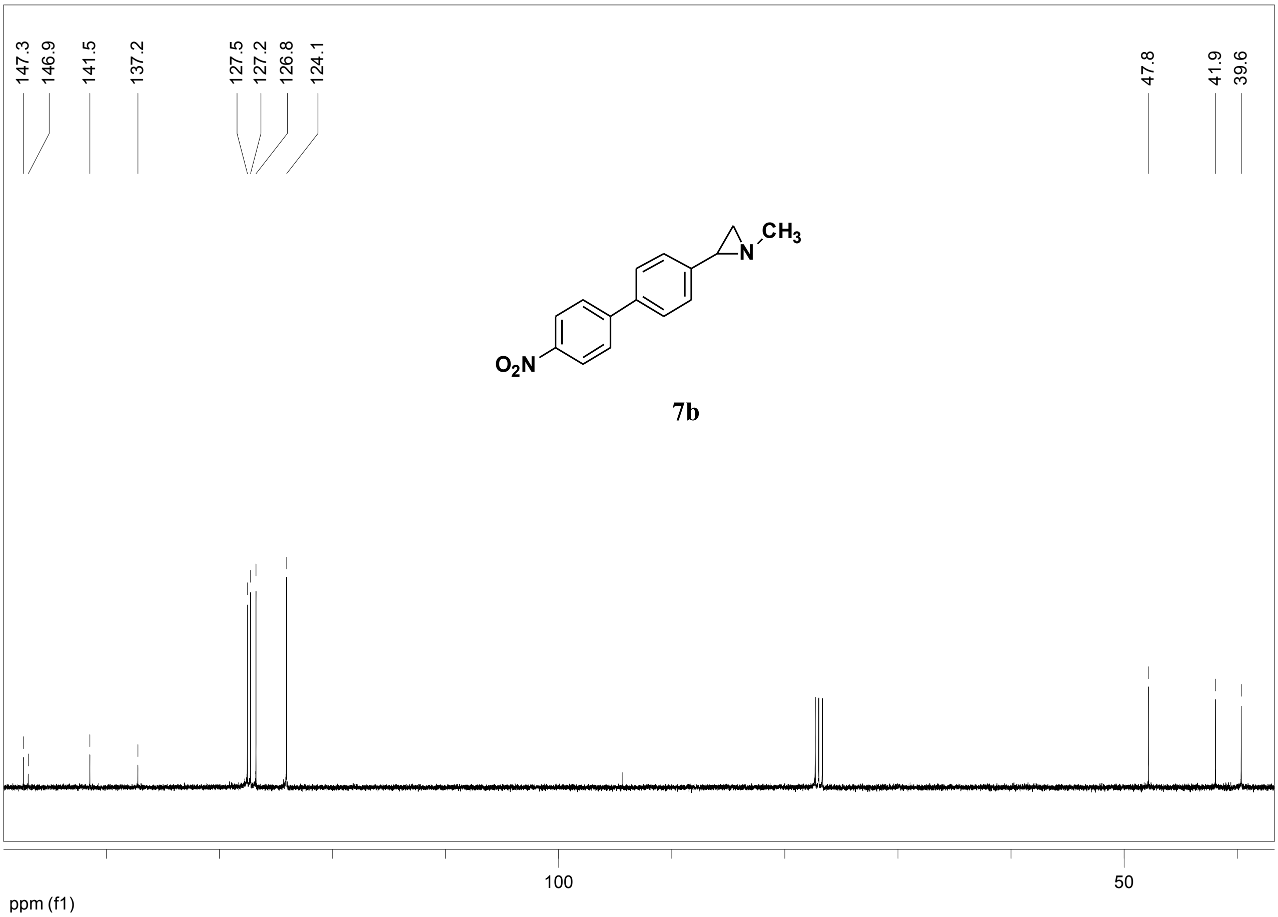
147.3
146.9
141.5
137.2
127.5
127.2
126.8
124.1
47.8
41.9
39.6
CH3
N
O2N
7b
100
50
ppm (f1)

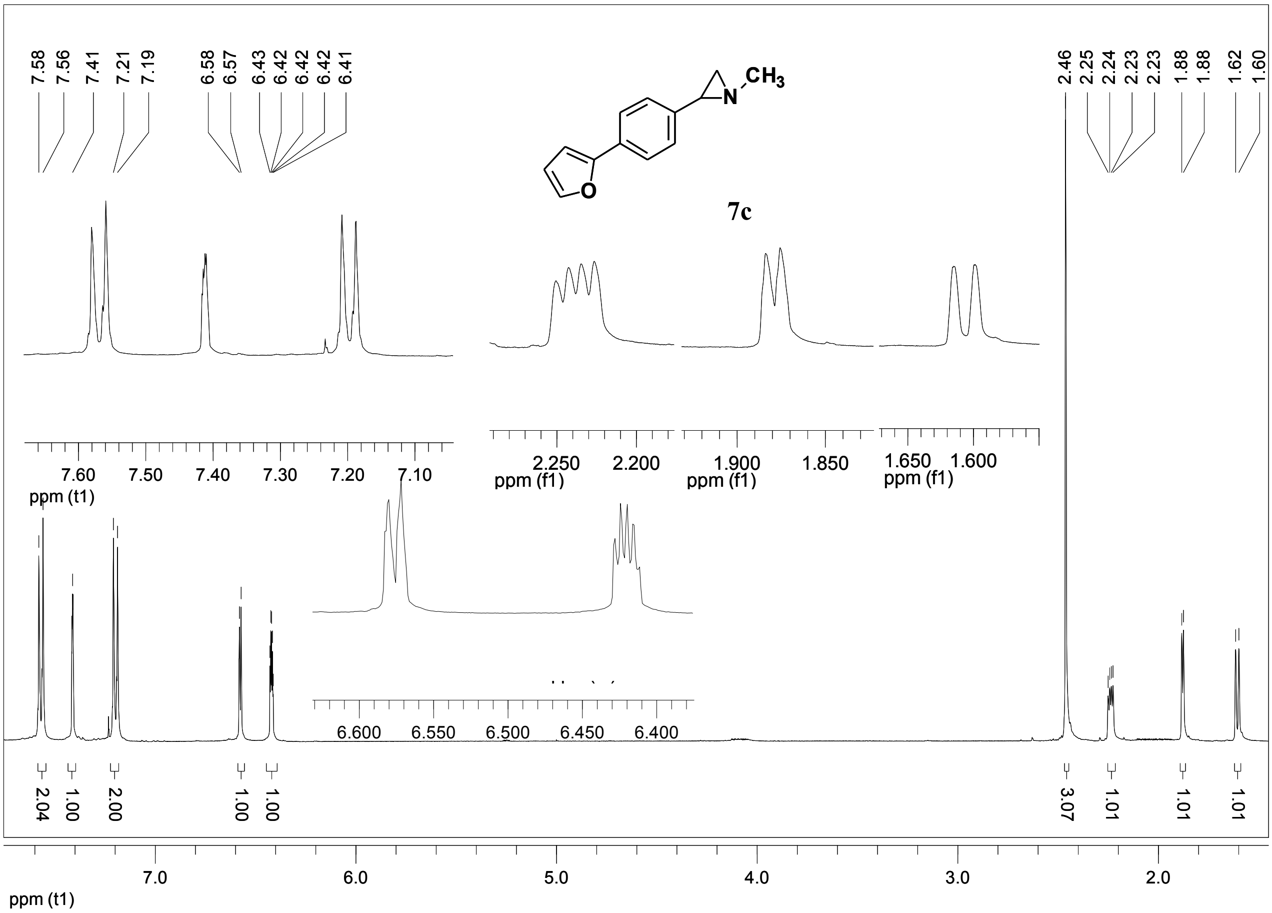
7.58
7.56
7.41
7.21
7.19
6.58
6.57
6.43
6.42
6.42
6.42
6.41
2.46
2.25
2.24
2.23
2.23
1.88
1.88
1.62
1.60
CH3
N
O
7c
7.60 7.50 7.40 7.30 7.20 7.10
ppm (t1)
2.250 2.200
ppm (f1)
1.900 1.850
ppm (f1)
1.650 1.600
ppm (f1)
6.600 6.550 6.500 6.450 6.400
2.04
1.00
2.00
1.00
1.00
3.07
1.01
1.01
1.01
7.0 6.0 5.0 4.0 3.0 2.0
ppm (t1)

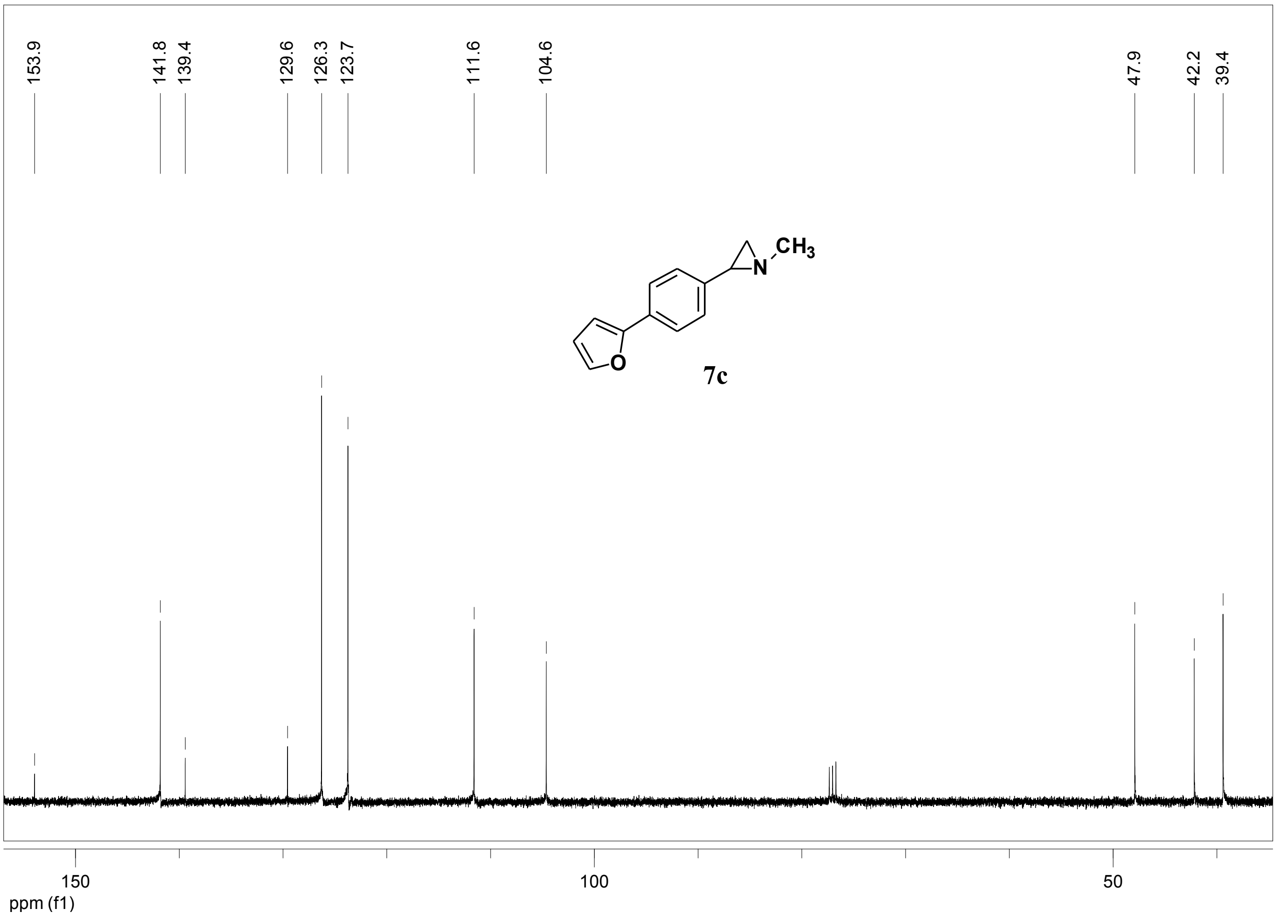
153.9
141.8
139.4
129.6
126.3
123.7
111.6
104.6
47.9
42.2
39.4
CH3
N
O
7c
150 100 50
ppm (f1)

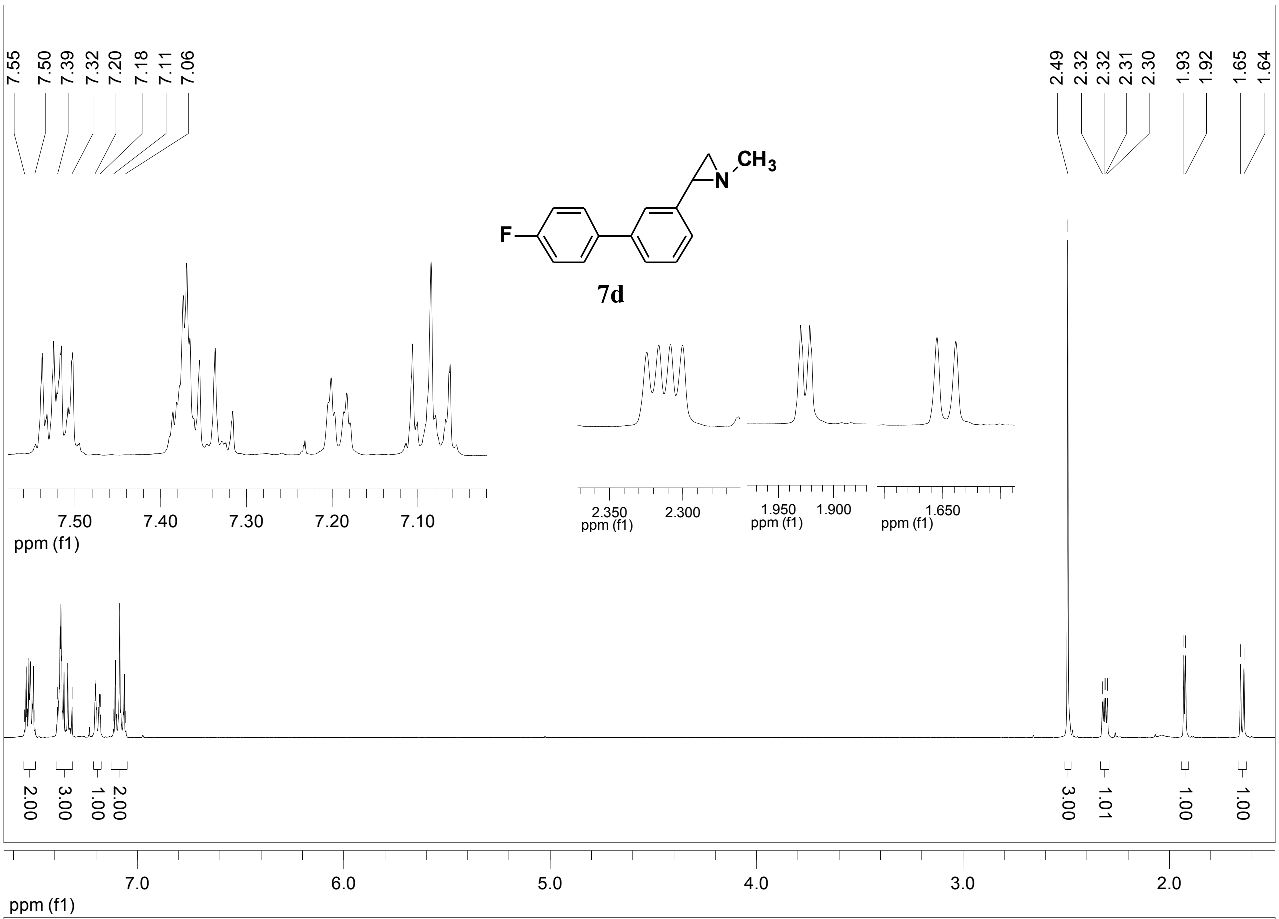
7.55 7.50 7.39 7.32 7.20 7.18 7.11 7.06
2.49 2.32 2.32 2.31 2.30 1.93 1.92 1.65 1.64
F
N
CH3
7d
7.50 7.40 7.30 7.20 7.10
ppm (f1)
2.350 2.300
ppm (f1)
1.950 1.900
ppm (f1)
1.650
ppm (f1)
2.00 3.00 1.00 2.00
3.00 1.01 1.00 1.00
7.0 6.0 5.0 4.0 3.0 2.0
ppm (f1)

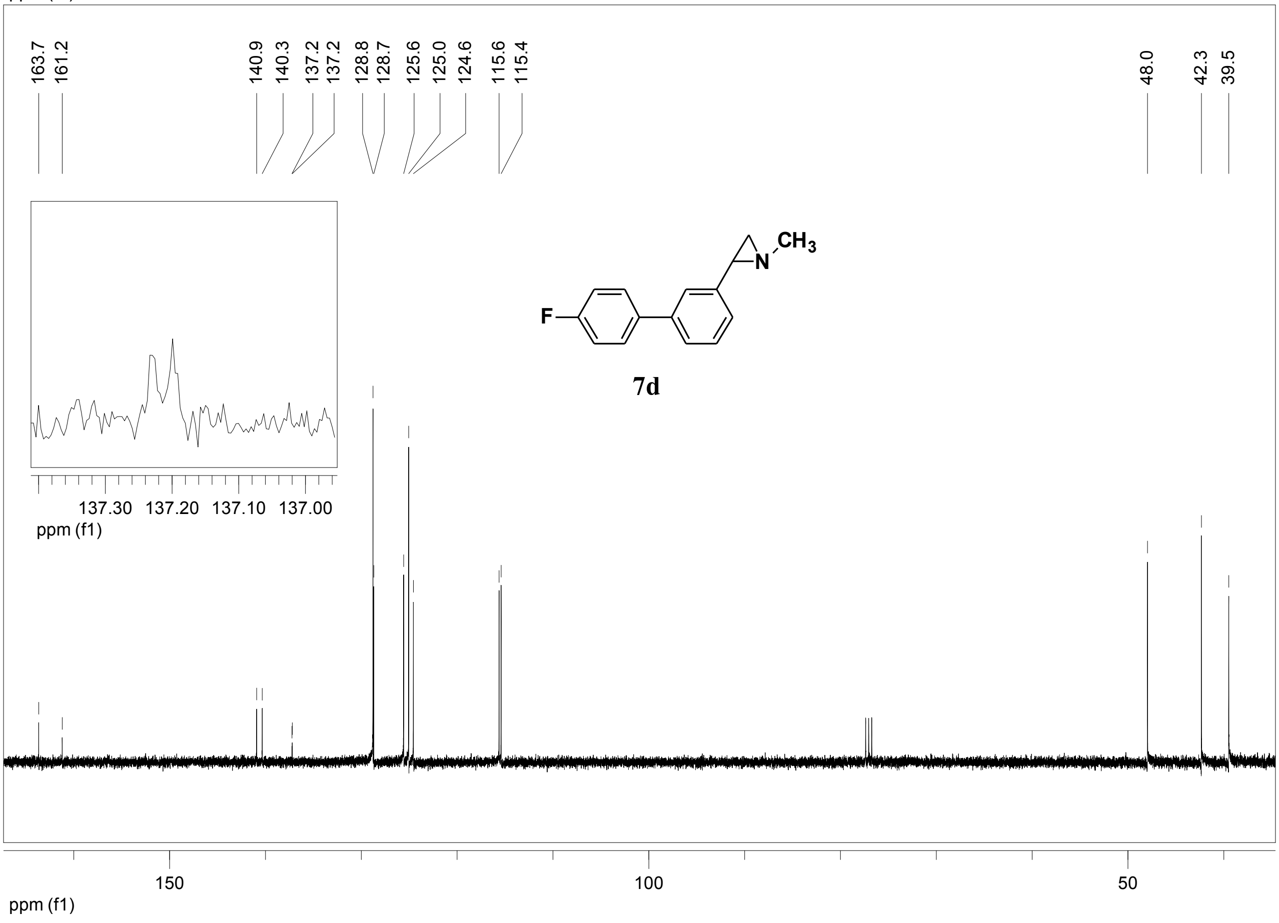
163.7 161.2
140.9 140.3 137.2 137.2 128.8 128.7 125.6 125.0 124.6 115.6 115.4
48.0 42.3 39.5
F
N
CH3
7d
137.30 137.20 137.10 137.00
ppm (f1)
150 100 50
ppm (f1)

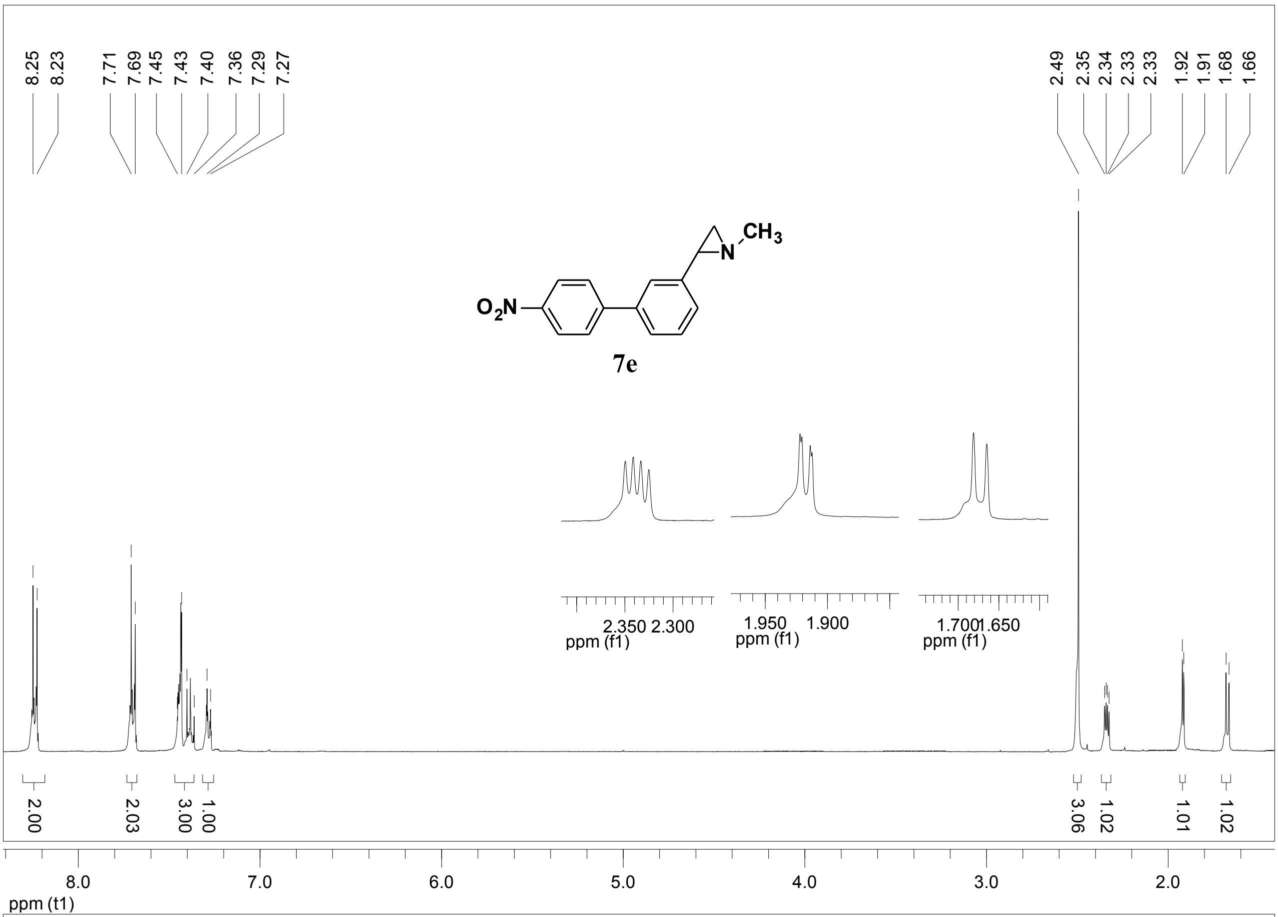
8.25
8.23
7.71
7.69
7.45
7.43
7.40
7.36
7.29
7.27
2.49
2.35
2.34
2.33
2.33
1.92
1.91
1.68
1.66
CH3
N
O2N
7e
2.350 2.300
ppm (f1)
1.950 1.900
ppm (f1)
1.700 1.650
ppm (f1)
2.00
2.03
3.00
1.00
3.06
1.02
1.01
1.02
8.0
7.0
6.0
5.0
4.0
3.0
2.0
ppm (t1)

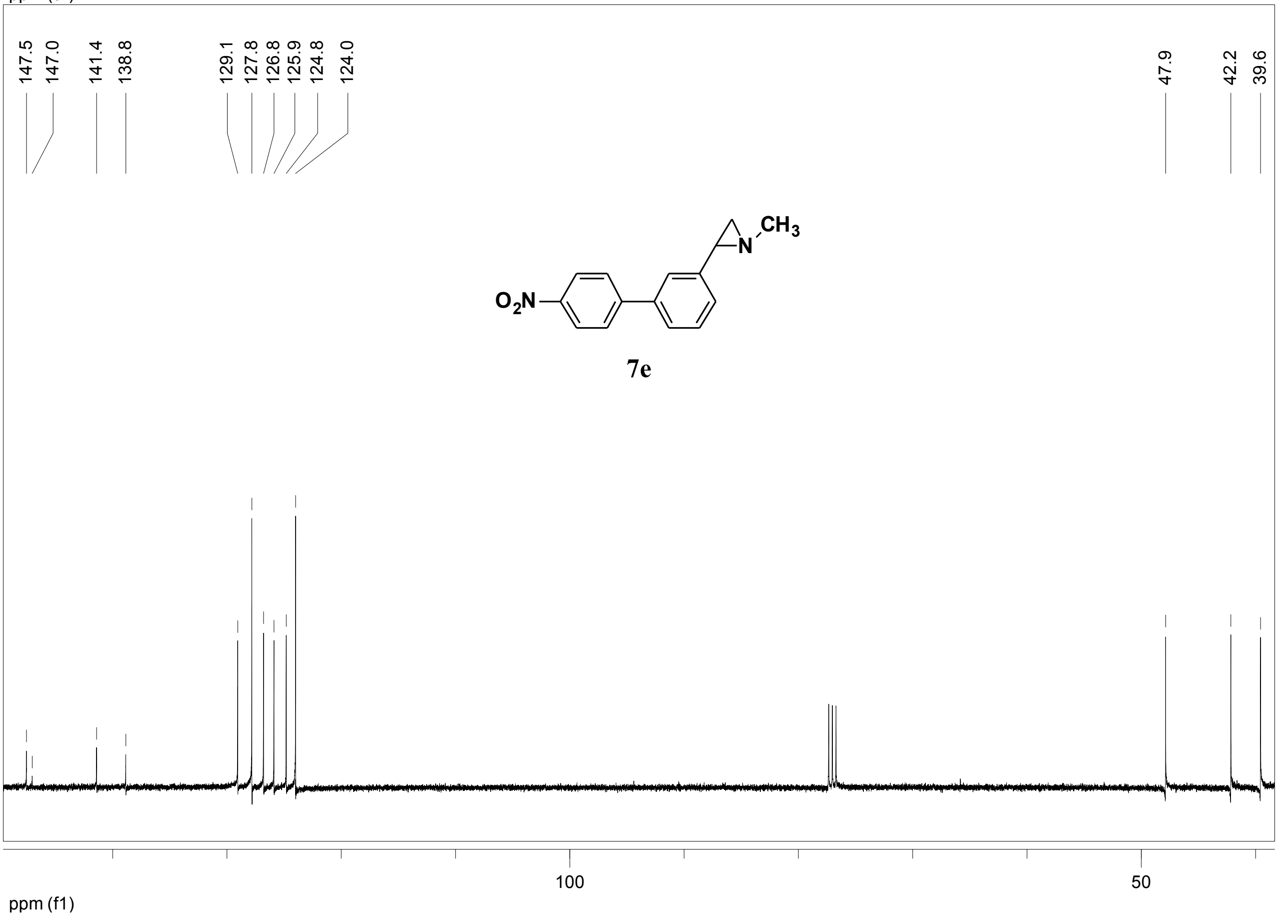
147.5
147.0
141.4
138.8
129.1
127.8
126.8
125.9
124.8
124.0
47.9
42.2
39.6
CH3
N
O2N
7e
100
50
ppm (f1)

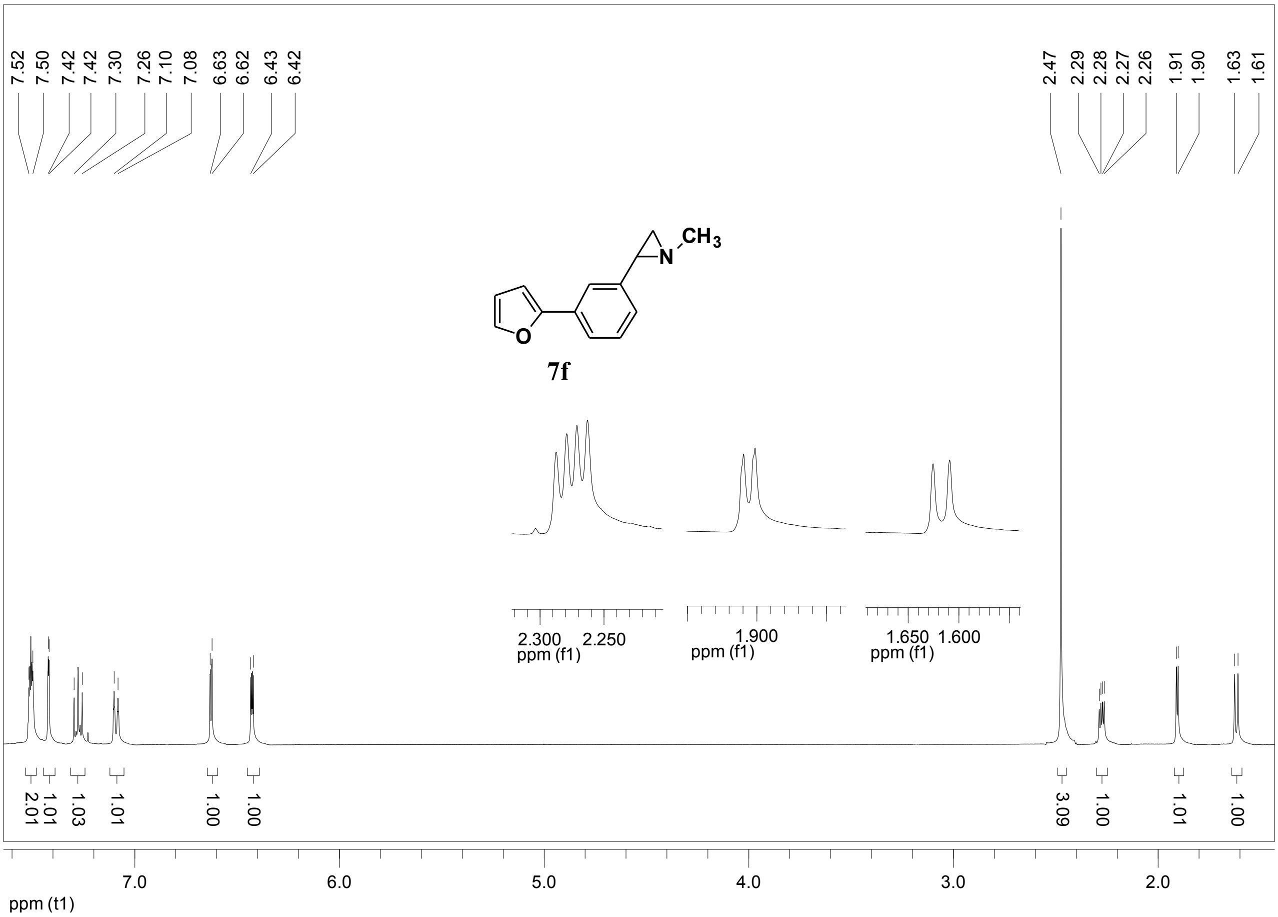
7.52
7.50
7.42
7.42
7.30
7.26
7.10
7.08
6.63
6.62
6.43
6.42
2.47
2.29
2.28
2.27
2.26
1.91
1.90
1.63
1.61
CH3
N
O
7f
2.300 2.250
ppm (f1)
1.900
ppm (f1)
1.650 1.600
ppm (f1)
2.01
1.01
1.03
1.01
1.00
1.00
3.09
1.00
1.01
1.00
7.0
6.0
5.0
4.0
3.0
2.0
ppm (t1)

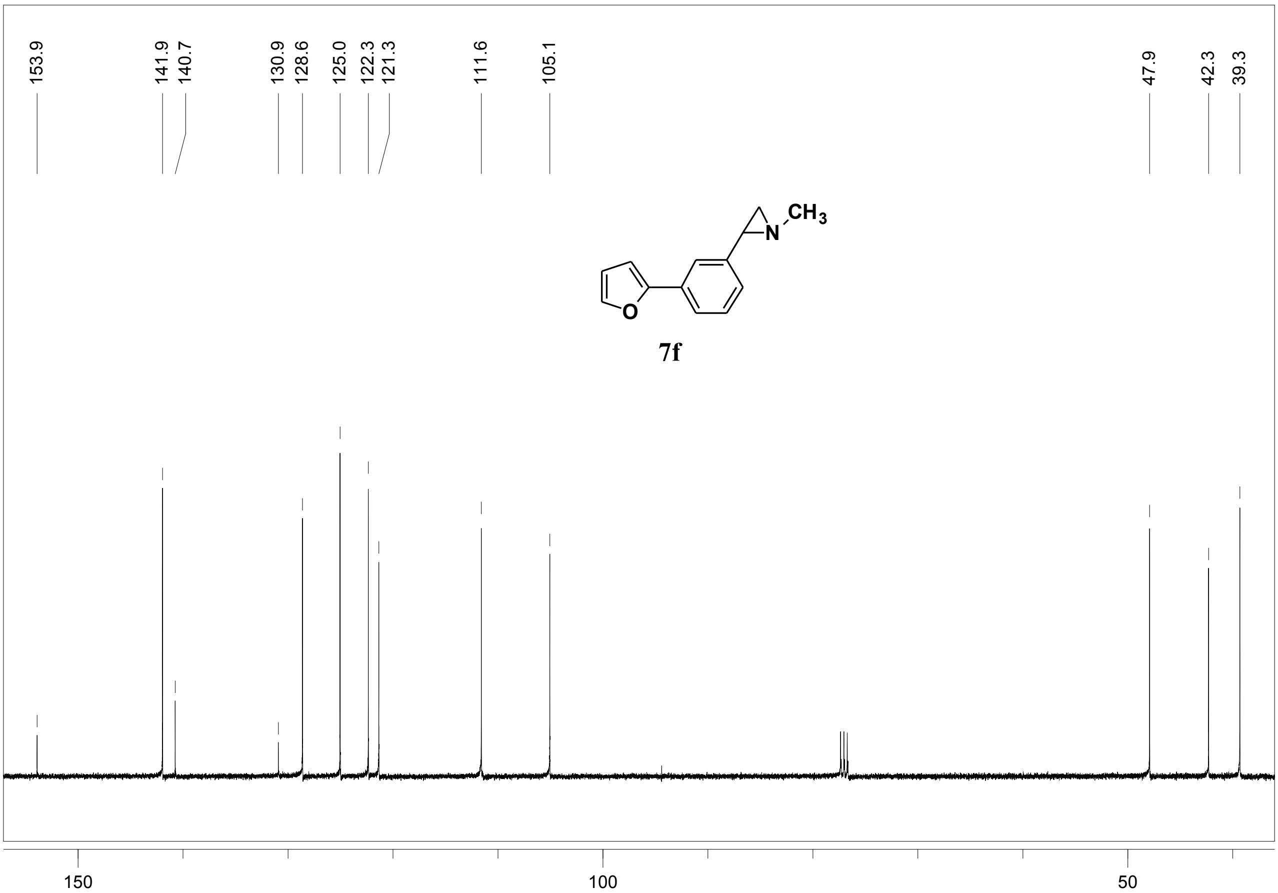
153.9
141.9
140.7
130.9
128.6
125.0
122.3
121.3
111.6
105.1
47.9
42.3
39.3
CH3
N
O
7f
150
100
50
ppm (f1)

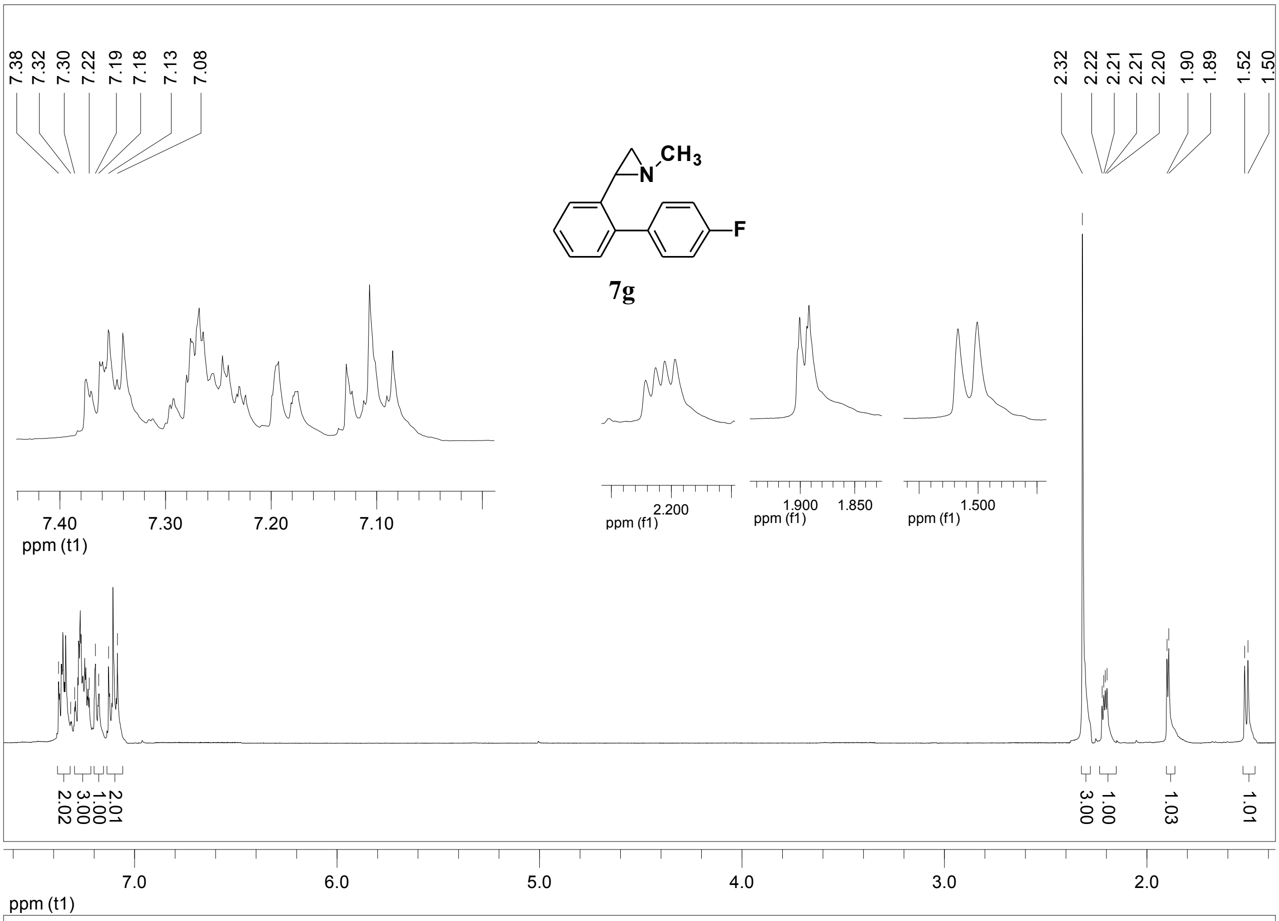
7.38
7.32
7.30
7.22
7.19
7.18
7.13
7.08
2.32
2.22
2.21
2.21
2.20
1.90
1.89
1.52
1.50
CH3
N
F
7g
7.40
7.30
7.20
7.10
ppm (t1)
2.200
ppm (f1)
1.900
1.850
ppm (f1)
1.500
ppm (f1)
2.02
3.00
1.00
2.01
3.00
1.00
1.03
1.01
7.0
6.0
5.0
4.0
3.0
2.0
ppm (t1)

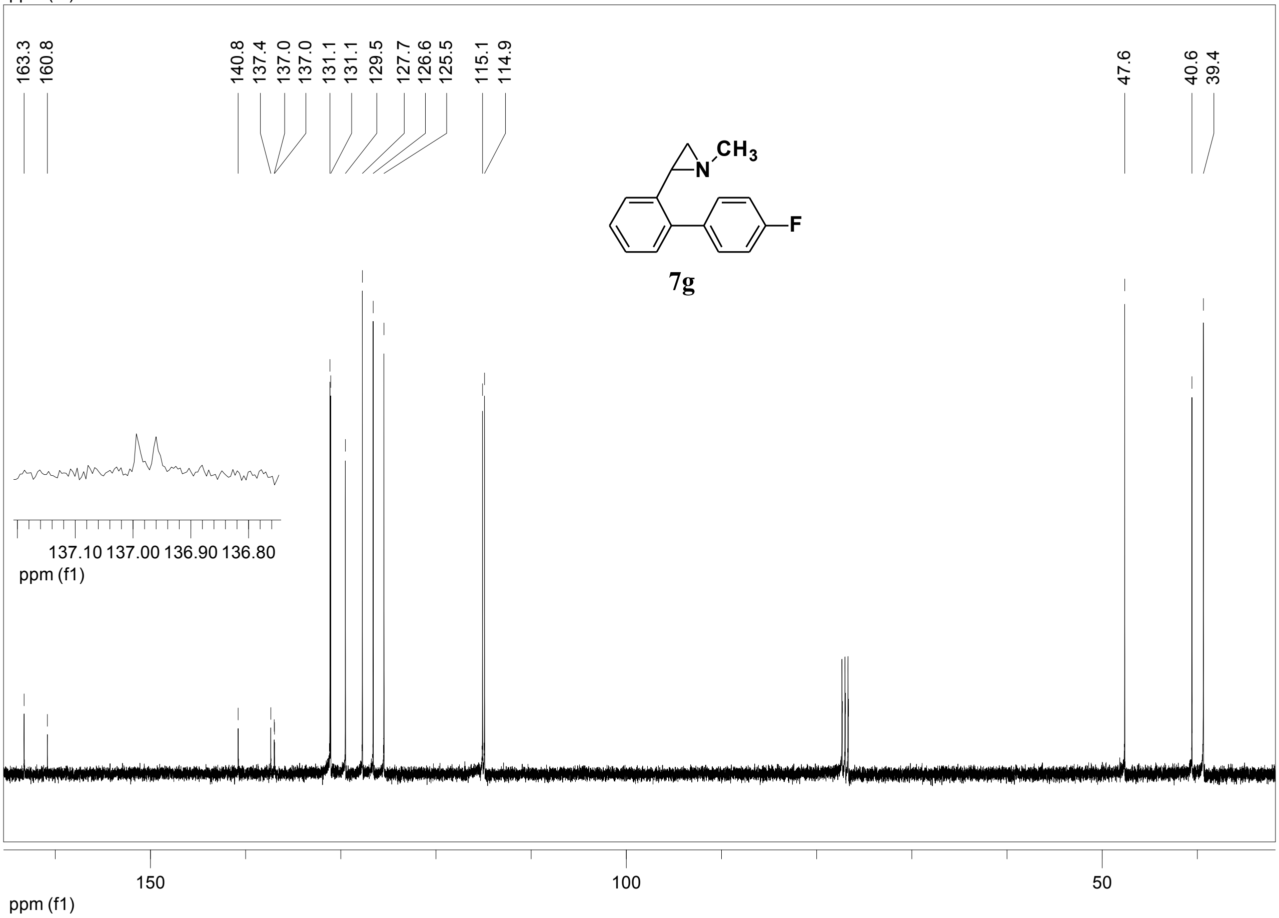
163.3
160.8
140.8
137.4
137.0
137.0
131.1
131.1
129.5
127.7
126.6
125.5
115.1
114.9
47.6
40.6
39.4
CH3
N
F
7g
137.10 137.00 136.90 136.80
ppm (f1)
150
100
50
ppm (f1)

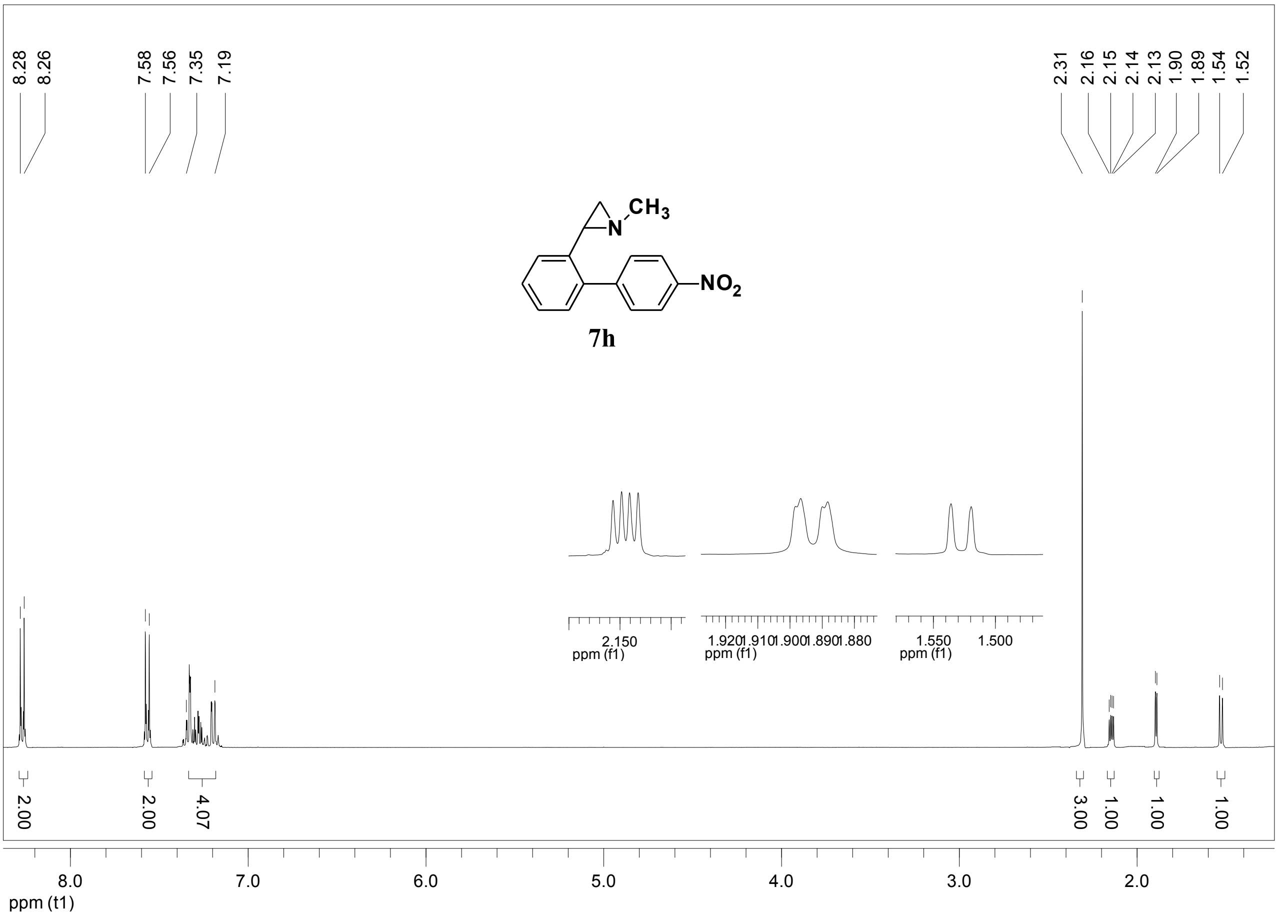
8.28
8.26
7.58
7.56
7.35
7.19
2.31
2.16
2.15
2.14
2.13
1.90
1.89
1.54
1.52
CH3
N
NO2
7h
2.150
ppm (f1)
1.920 1.910 1.900 1.890 1.880
ppm (f1)
1.550 1.500
ppm (f1)
2.00
2.00
4.07
3.00
1.00
1.00
1.00
8.0
7.0
6.0
5.0
4.0
3.0
2.0
ppm (t1)

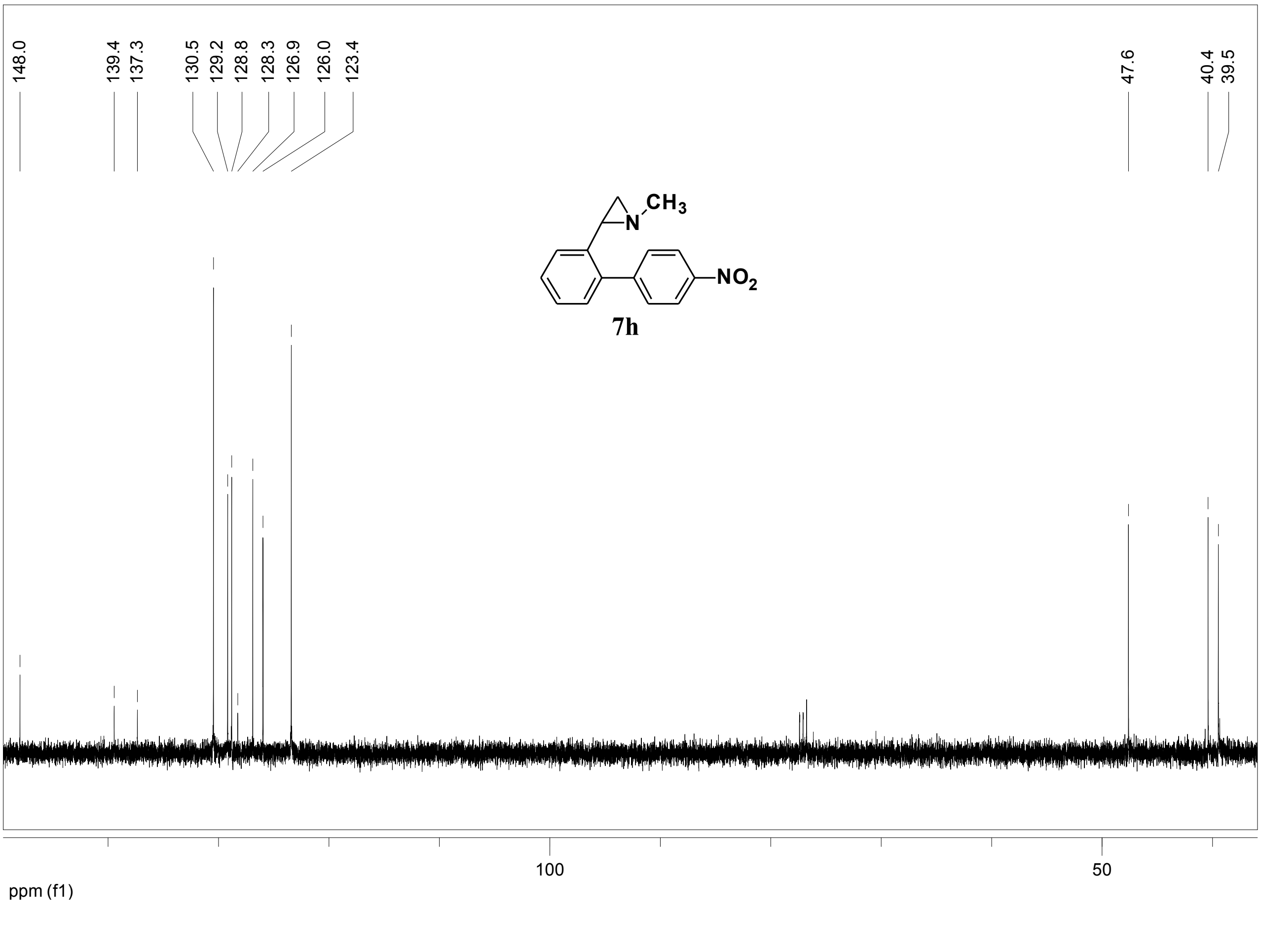
148.0
139.4
137.3
130.5
129.2
128.8
128.3
126.9
126.0
123.4
47.6
40.4
39.5
CH3
N
NO2
7h
100
50
ppm (f1)

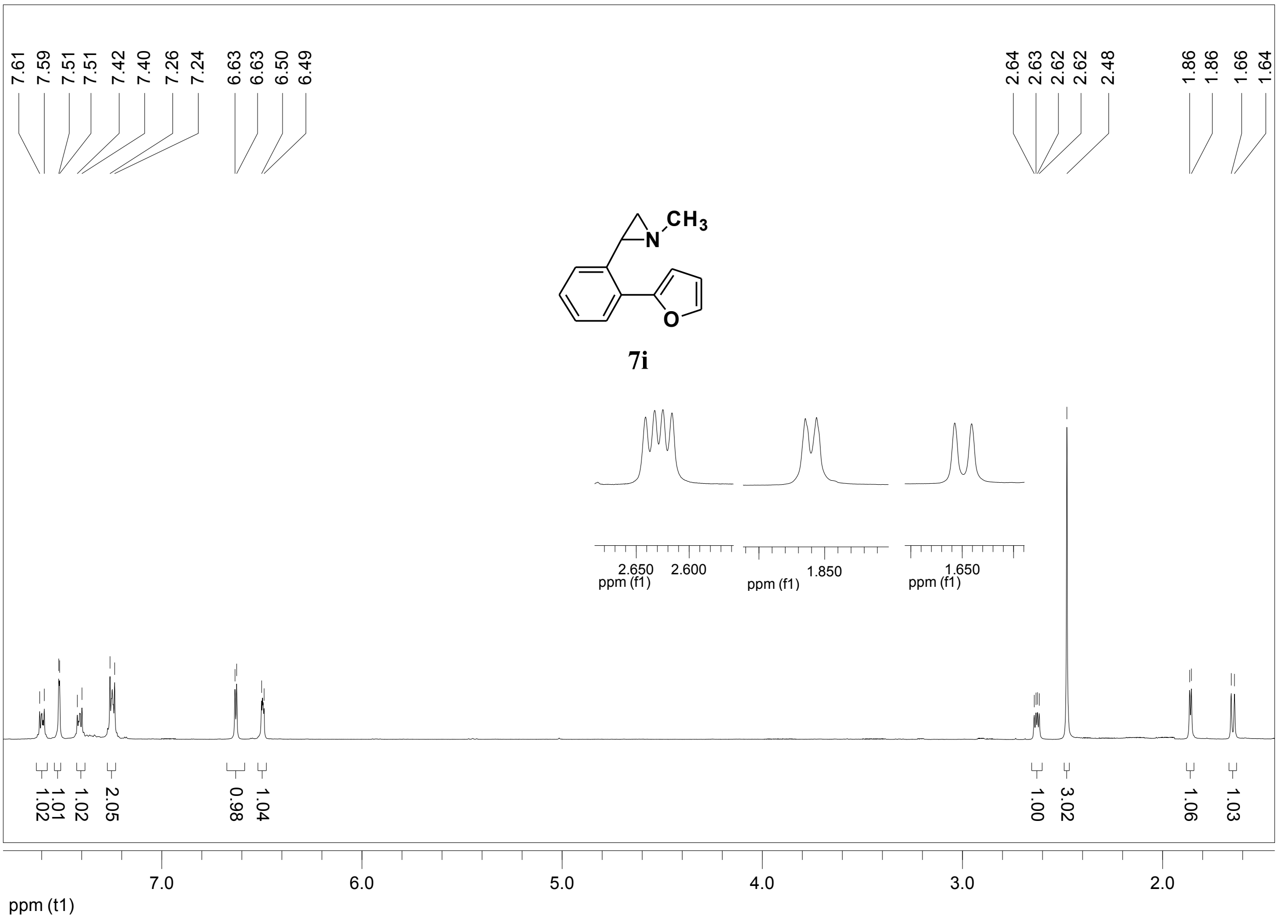

7.61 7.59 7.51 7.51 7.42 7.40 7.26 7.24 6.63 6.63 6.50 6.49
2.64 2.63 2.62 2.62 2.48
1.86 1.86 1.66 1.64
CH3
N
O
7i
2.650 2.600
ppm (f1)
1.850
ppm (f1)
1.650
ppm (f1)
1.02 1.01 1.02 2.05 0.98 1.04 1.00 3.02 1.06 1.03
7.0 6.0 5.0 4.0 3.0 2.0
ppm (t1)

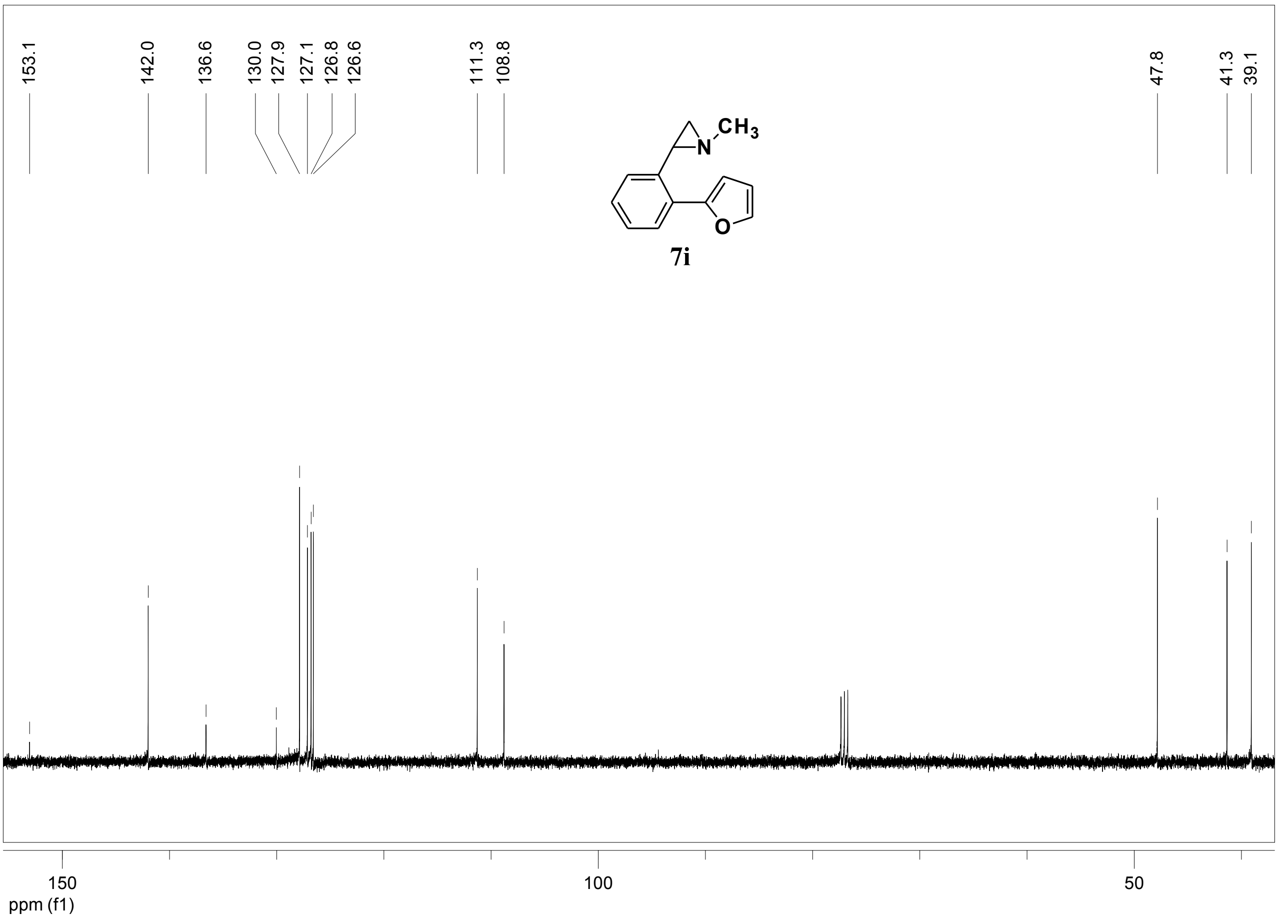

153.1 142.0 136.6 130.0 127.9 127.1 126.8 126.6 111.3 108.8 47.8 41.3 39.1
CH3
N
O
7i
150 100 50
ppm (f1)
